# *cis-* and *trans-*elements for the transcriptional regulation of sugar responsive genes: from current knowledge to future applications

**DOI:** 10.1007/s11103-025-01642-1

**Published:** 2025-11-04

**Authors:** María-Isabel Cruz-López, Josefat Gregorio, Elizabeth Cordoba

**Affiliations:** 1 Departamento de Biología Molecular de Plantas, Instituto de Biotecnología, Universidad Nacional Autónoma de México, Av. Universidad #2001, Col. Chamilpa, C.P. 62210 Cuernavaca, Morelos México; 2 Secretaría de Ciencia, Humanidades, Tecnología e Innovación-Comisión Nacional del Agua, Av. Insurgentes Sur 1582, Col. Crédito Constructor, Del. Benito Juárez, C.P. 03940 Ciudad de México, México

**Keywords:** *cis*-regulatory elements, *trans*-regulatory elements, Sugar signaling, Biotechnology, Gene regulation

## Abstract

Sugar metabolism in plants is highly dynamic throughout their life cycle, driven by the continuous production, accumulation, and distribution of these molecules along the plant body. To cope with fluctuating sugar levels during their life cycle, plants have developed mechanisms to sense and respond to these changes accordingly. Noteworthy, sugars not only fulfill metabolic roles, but also act as signaling molecules that regulate plant growth and development. Of the array of sugar responses, their influence on gene expression is particularly significant, as it impacts a wide range of physiological processes, including key economic traits of plants. However, despite the broad regulatory role of sugars in gene expression, the transcriptional mechanisms behind their regulation remain largely unknown. Among the many sugar-regulated genes in plants, efforts have been focused on identifying *cis-*regulatory elements (CREs) and *trans*-regulatory factors (transcription factors, TFs) involved in gene sugar responsiveness at transcriptional level, but only some have been experimentally confirmed. Therefore, this review outlines those approaches used for identifying sugar CREs and TFs, along with an updated compilation of the elements associated with glucose and sucrose signaling transcriptional responses. In addition, the evolutionary conservation of these regulatory elements in different plant species is addressed, highlighting those with potential biotechnological applications. In summary, the gathering of this information has the purpose of updating our current knowledge regarding the mechanism of how sugars exert its effect on gene expression. This understanding is essential for advancing in the manipulation of these regulatory elements to improve key traits in economically valuable plants, such as oil and sugar accumulation, crop yield, and fruit quality.

## Key message

This review describes the identified transcription responsive regulatory elements involved in sugar signaling in plants, highlighting their potential biotechnological applications for enhancing desirable traits in plants.

## Introduction

The sugar metabolism in plants is dynamic throughout their life cycle; consequently, plants have developed mechanisms to sense and adapt to fluctuating sugar levels. This adaptability is denoted by the regulatory role of sugars, which function as signaling molecules that control diverse developmental processes such as embryogenesis, flowering, fruit and storage organ development, and root architecture (Sheen 1990; Koch 1996; Jang et al. 1997; Sheen et al. 1999; Gibson 2005). Until now, the sugars described as having signaling functions in plants, independently of their metabolic role, are Glc (glucose), Fru (fructose), Suc (sucrose) and Tre6P (the phosphorylated form of trehalose) (Sheen 1990; Chiou and Bush 1998; Umemura et al. 1998; Zhang et al. 2009; Cho and Yoo 2011; Xiong et al. 2013; Yadav et al. 2014).

**Fig. 1:**
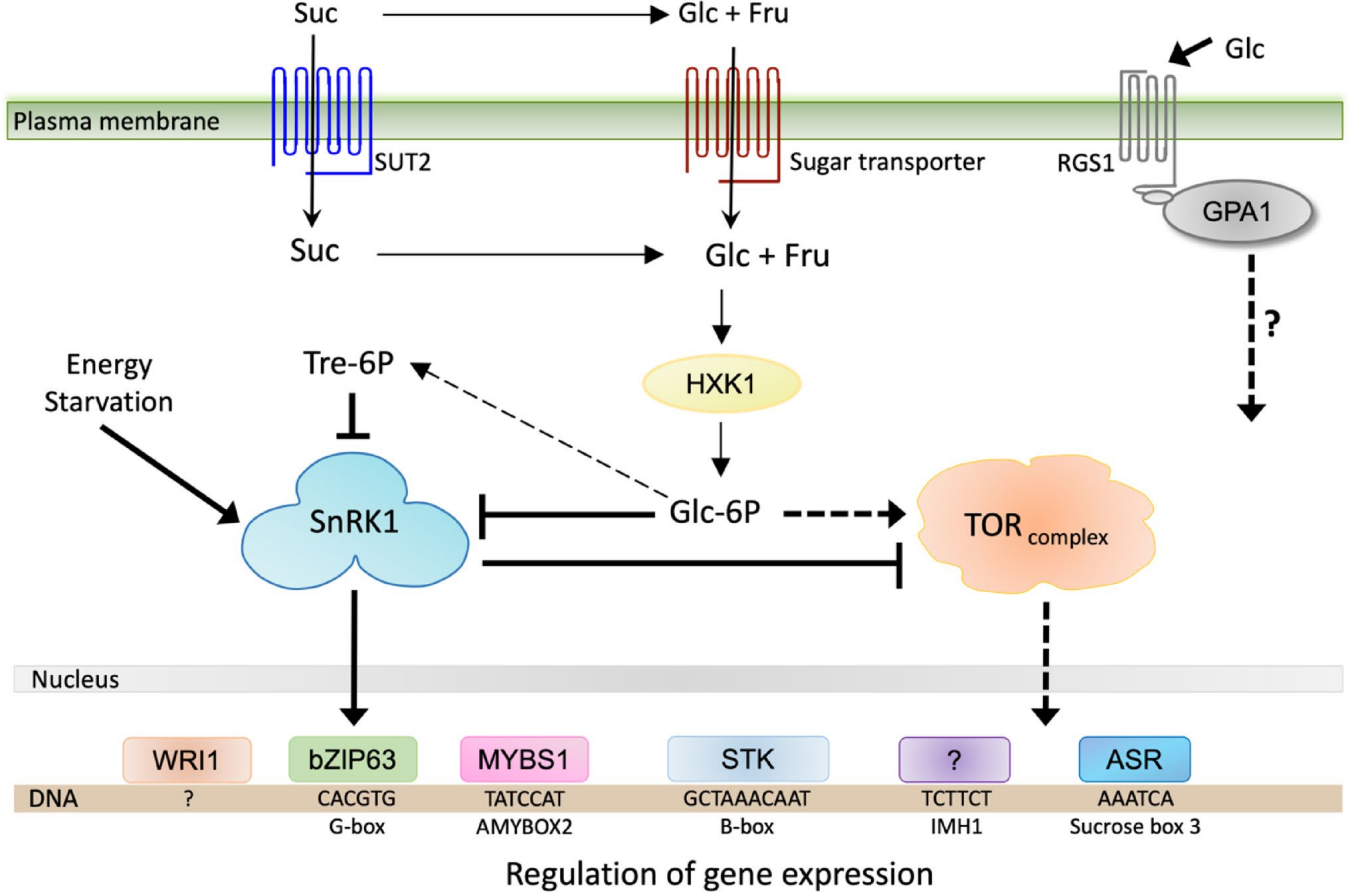
Model of sugar signaling in plants. The Hexokinase (HXK1), SUT2, and G-protein coupled receptor signaling (involving RGS1 and GPA1), are involved in sugar-sensing. The SnRK1 complex is activated by energy starvation signaling and inhibited by Glc-6-phosphate (Glc-6P) and trehalose-6-phosphate (Tre-6P), whereas TOR is activated by nutrient availability (Glc-6P) and regulated by SnRK1. In the nucleus, several transcription factors (TFs) such as bZIP63, MYBS1, STK, and ARR regulate sugar-responsive gene expression by binding to specific *cis*-regulatory elements (CREs). In some cases, the CRE involved in sugar regulation (e.g., IMH1) has been identified, but its corresponding TF remains unknown. Conversely, certain TFs (e.g., WRI1) have been characterized, although the CRE they target has not yet been determined. Processes related to regulatory sugar signaling and those related to transport and metabolism are indicated with thick or thin lines, respectively. Lines ending in an arrowhead correspond to positive regulation and vertical lines to negative regulation. Dotted lines indicate that several steps are involved, while solid lines indicate direct effect. The name for the corresponding CRE is below its sequence

Given the regulatory role of sugars as signaling molecules, efforts have been made to unravel the molecular mechanisms underlying the signaling cascades they trigger, leading to the identification of key components (Fig. 1). In plants, a few sugar sensors have been recognized, including the Glc sensor HEXOKINASE1 (HXK1), the putative membrane sugar sensors SUCROSE TRANSPORTER 2 (SUT2), and REGULATOR OF G-PROTEIN SIGNALING 1 (RGS1), and the sensors of energy status, the kinase complexes Snf1-RELATED PROTEIN KINASE 1 (SnRK1), and TARGET OF RAPAMYCIN (TOR) (Fig. 1) (Jang et al. 1997; Barker et al. 2000; Baena-González et al. 2007; Polge and Thomas 2007; Grigston et al. 2008). Evaluation of mutants and transgenic overexpressors on the glycolytic enzyme HXK1 revealed its role as a Glc-sensor in plants, independent of its metabolic function (Jang et al. 1997). SUT2, which shares features with the yeast sugar sensor SNF3 and RGT2, is proposed to regulate *SUT1* and *SUT4* gene expression in sieve cells during sugar loading (Barker et al. 2000). The mutant and overexpressing *RGS1* lines of *Arabidopsis* exhibited altered Glc-sensitivity during seed germination and seedling development (Grigston et al. 2008). Meanwhile, SnRK1 and TOR integrate energy signaling, responding to nutrient starvation or availability, respectively (Baena-González et al. 2007; Polge and Thomas 2007; Xiong et al. 2013; Dobrenel et al. 2016). All these molecular components act in a concerted way, and ultimately regulate gene expression by modulating the activity of TFs, which control the expression of target genes. In this regard, global gene expression analyses in *Arabidopsis* have identified distinct sets of genes that are either induced or repressed in response to different sugar signaling molecules (Kunz et al. 2014). For example, Glc upregulated 444 genes and downregulated 534 (Price et al. 2004). Similarly, Suc induced 900 and repressed 748 genes, whereas trehalose induced 56 and repressed 9 genes (Thum et al. 2004; Aghdasi et al. 2012). According to their function, the sugar-regulated genes are involved in a wide range of biological roles, from stress response and cellular metabolism to those involved in signaling and gene regulation (Price et al. 2004; Thum et al. 2004; Aghdasi et al. 2012; Kunz et al. 2014). These findings highlight the importance of transcriptional regulation as a central component of sugar responses.

To better understand how these extensive transcriptional changes are achieved, it is necessary to consider the molecular framework of transcription regulation. In general, this process involves the interaction between three elements: (i) the core promoter region, housing the TATA box where the preinitiation complex (PIC) is assembled; (ii) the proximal promoter region, containing CREs, represented by short DNA sequences (6–12 bases) that are recognized by (iii) particular regulatory factors, namely TFs (Nath et al. 2006; Latchman 2011). In response to stimuli, specific TFs bind to their corresponding CREs to regulate transcription. Then, TFs interact with the RNA polymerase II, subsequently resulting in the activation or repression of transcription. Some TFs have dual function given their interaction with both CREs and other TFs, making their activity context-dependent (Stracke et al. 2001; Fuda et al. 2009; Hernandez-Garcia and Finer 2014).

Despite the large number of sugar-regulated genes, the underlying transcriptional mechanism(s) remain elusive. Studies have concentrated on identifying CREs and TFs, but within the extensive array of sugar regulated genes, only a few regulatory components have been functionally characterized. Most of these elements were identified analyzing sugar-responsive genes, through classical molecular techniques; however, cutting-edge technological advances, including next-generation sequencing (NGS), have enabled to carry out global studies in this topic. This review outlines the techniques employed, compiles the identified CREs and TFs involved in sugar responses, explores their conservation in some plant species, and discusses how these regulatory elements could be exploited to modulate key traits in economically important plants such as oil and sugar accumulation, crop yield (starch), color, and fruit harvest time (Mao et al. 2018; Durán-Soria et al. 2020).

## Unveiling cis-regulatory elements in sugar signaling pathways

The identification of CREs involved in sugar responses in plants has primarily relied on promoter deletion analysis, a widely used approach to dissect regulatory regions controlling gene expression. This method involves identifying and isolating the promoter region of a sugar-responsive gene, fusing it to a reporter gene (e.g., β-glucuronidase, luciferase or a fluorescent protein) and assessing its transcriptional activity in response to sugar stimuli. Next, different fragments of the promoter are deleted until the sugar transcription-controlling region is delimited. These experiments can be performed through transient expression (e.g., protoplast assays) or stable transformation systems (e.g., transgenic plants) (Xu et al. 2013; Schmitz et al. 2022). However, although this approach effectively narrows down regulatory regions, it does not allow identification of the specific CRE(s) responsible for exerting the positive or negative effect observed on the gene reporter’s activity output. To address the latter, inserting of mutations or deleting specific motifs within the delimited region, serve as complementary approaches that facilitate a closer identification of the CRE or combination of CREs involved in controlling the transcriptional output (Hwang et al. 1998; Xu et al. 2013; Galli et al. 2020; Schmitz et al. 2022). Many sugar-responsive CREs listed in Table 1 were identified through the promoter deletion analysis, often in combination with other molecular techniques.

**Table 1:** *Cis*-regulatory elements associated with sugar-regulated responses in plants

CRE	Sequence	Gene	Protein	Species	Method	References
SP8A	ACTGTGTA	*gSPO-A1* *gβ-Amy*	Sporamin A β-amylase	*Ipomoea batatas*	b, c b, c	Ishiguro and Nakamura 1992
SP8a-like	ACTGTGTT	*iso1*	Isoamylase	*Hordeum vulgare*	c, d	Sun et al. 1999
SP8b	TACTATT	*gSPO-B1*	Sporamin B	*Ipomoea batatas*	b, c	Ishiguro and Nakamura 1992 Maeo et al. 2001 Li et al. 2006
		*gβ-Amy*	β-amylase		b, d	
		*β-Amy*	β-amylase	*Arabidopsis*	a	
GARE2OSREP1	TAACGTA^+^		Secondary metabolism		m	
AT-rich sequence	53 bases fragment	*PI-II*	Proteinase inhibitor II	*Solanum tuberosum*	a, d	Kim et al. 1991 Liu et al. 1990
		*pat21*	Patatin-1		a, d	
B-box	GCTAAACAAT	*pat21*	Patatin-1	*Solanum tuberosum*	a, b, c	Grierson et al. 1994 Zourelidou et al. 2002
		PS20*	Patatin*		d	
		B33*			d	
SURE-1	AATAGAAAA	*pat21*	Patatin-1	*Solanum tuberosum*	a, b, c	Grierson et al. 1994 Atanassova et al. 2003 Jiaxing et al. 2018 Sun et al. 2003
		*VvHT1*	Hexose transporter 1	*Vitis vinifera*	a, c, d	
		*PpHT*	Hexose transporter	*Prunus persica*	d, g	
		*iso1*	Isoamylase	*Hordeum vulgare*	c, d	
SURE-2	AATACTAAT	*pat21*	Patatin-1	*Solanum tuberosum*	a, b, c	Grierson et al. 1994 Mufarrege et al. 2009
		*AtCOX6b-1*	Cytochrome c oxidase subunit	*Arabidopsis*	d, f	
		*AtCOX6b-2*			d, f	
		*AtCOX6b-3*			d, f	
AMYBOX2 Sucrose repressor	TATCCAT CTTATCCA	*αAmy3*	α-amylase	*Oryza sativa*	a, c, f m	Hwang et al. 1998; Lu et al. 1998; Toyofuku et al. 1998 Geisler et al. 2006
AMYBOX2-like Sucrose repressor	CTTATCC CTTATCCA	*AS*	Asparagine synthase	*Asparagus officinalis*	a, d, c m	Winichayakul et al. 2004 Geisler et al. 2006
G-box related	CACGT	*αAmy3*	α-amylase	*Oryza sativa*	a, f	Hwang et al. 1998; Lu et al. 1998; Toyofuku et al. 1998
G-box	CACGTG	*rbcS2*	1,5-bisphosphate carboxylase/oxygenase small subunit	*Phaseolus vulgaris*	a, f	Urwin and Jenkins 1997 Li et al. 2006
QARBNEXTA	AACGTGT				m	
LRENPCABE	ACGTGGCA		Carbohydrate, lipid and amino acid metabolism		m	
G-box like	CACATG	*rcbS2*	1,5-bisphosphate carboxylase/oxygenase small subunit	*Phaseolus vulgaris*	a	Urwin and Jenkins 1997 Wawrzynska et al. 2022
	AGATGCACAT	*PAP1*	Production of anthocyanin pigment1	*Arabidopsis*	e, f, h, i	
GC-box DRECRTCOREAT	CGACG RCCGAC	*αAmy3*	α-amylase Abiotic stress	*Oryza sativa*	a, f m	Hwang et al. 1998; Lu et al. 1998 Li et al. 2006
IMH1	TCTTCT	*MS*	Malate synthase	*Cucumis sativus*	a, d, c	Graham et al. 1994; Sarah et al. 1996 Geisler et al. 2006
SUC-6	GAANGAGANGA^+^				m	
IMH2	CCCA(C/A)CCT	*MS*	Malate synthase	*Cucumis sativus*	a, d, c	Reynolds and Smith 1995 Sarah et al. 1996 Li et al. 2006 Geisler et al. 2006
		*ICL*	Isocitrate lyase		a, d	
TELOTEF	AAACCCTAA		Ribosomal proteins and protein synthesis		m	
SUC/ROS-1	CCCGCCTC				m	
SUC/ROS-2	CCTGCCTC				m	
AMMORESIIUDCRNIA1	GGWAGGGT^+^		Nucleotide metabolism		m	
Sucrose box 3	AAATCA	*PpHT*	Hexose transporter	*Prunus persica*	d, g	Jiaxing et al. 2018 Atanassova et al. 2003 Jia et al. 2016 Li et al. 2006
		*VvHT1*		*Vitis vinifera*	a, c, d	
		*SlHT1*		*Solanum lycopersicum*	d, e	
		*FaHT1*		*Fragaria x ananassa*	d, e	
		*FaHT2*			d	
		*FaHT3*			d	
EVENINGAT	AAAATATCT		Jasmonate synthesis and abiotic stress		m	
S-box	CACCTCCA	*rcbS1* *rbcS2*	1,5-bisphosphate carboxylase/oxygenase small subunit	*Nicotiana tabacum*	d, f	Acevedo-Hernandez et al. 2005 Urwin and Jenkins 1997
				*Lycopersicon esculentum*	d	
				*Solanum tuberosum*	d	
				*Petunia hybrida*	d	
				*Phaseolus vulgaris*	d	
CMSRE-1	TGGACGG	*gSPO-A1*	Sporamin A	*Ipomoea batatas*	a, f	Morikami et al. 2005 Maeo et al. 2001 Mita et al. 1995 Geisler et al. 2006 Li et al. 2006
		*gβ-Amy*	β-amylase		a, f	
		*β-Amy*	β-amylase	*Arabidopsis*	d, g	
SUC/ROS-6 SUC/ROS-7 SUC/ROS-9	GATGGATG				m	
Core GGCA						Geisler et al. 2006 Li et al. 2006
SUC/ROS-7	CAGGCAGG				m	
SUC/ROS-9	GAGGCAGG				m	
E2FBNTRNR	GCGGCAAA		Protein synthesis		m	
CMSRE-2	AGACACCGTAAGTGTTCCA	*gSPO-A1*	Sporamin A	*Ipomoea batatas*	a	Morikami et al. 2005
CE1	CACCG	*ADH1*	Alcohol dehydrogenase1	*Arabidopsis*	c, d, g	Niu et al. 2002 Arroyo et al. 2003 Bossi et al. 2009
		*PC*	Plastocyanin		d	
		*APL3*	ADP-Glucose pyrophosphorylase subunit		d	
		*ABI4*	ABA INSENSITIVE 4		c, d, i, e	
		*ABI5*	ABA INSENSITIVE 5		c, d, i, e	
		*SBE2.2*	Starch branching enzyme 2.2		c, d, i, e	
ATCATT	ATCATT	*AtCOX5b-1*	Cytochrome b oxidase subunit	*Arabidopsis*	a, c, i	Comelli et al. 2009
Site II element	TGGGCC	*AtCOX5b-2* *Cytc-2*	Cytochrome b oxidase subunit	*Arabidopsis*	a, d, c	Comelli and Gonzalez 2009 Welchen et al. 2009
Site II element-like	TGGGTC	*AtCOX5b-2*	Cytochrome b oxidase subunit	*Arabidopsis*	a, d, c	Comelli and Gonzalez 2009
Core GCCT	GCCT	*AtPGR*	Plasma membrane Glc-regulated	*Arabidopsis*	a, c, h, i	Chung et al. 2016
GAGA	GAGA	*AtPGR*	Plasma membrane Glc regulated	*Arabidopsis*	a, c, h, i	Min et al. 2017 Geisler et al. 2006
SUC-6	GAANNGA				m	
W-box	TGACT	*ClTST2*	Tonoplast sugar transporter	*Citrullus lanatus*	a, c, h, j, l	Ren et al. 2018 Cheng et al. 2018 Wei et al. 2019
		*CmTST2*		*Cucumis melo*	d	
		*HpINV2*	Invertase	*Hylocereus polyrhizus*	c, g	
		*HpSuSy1*	Sucrose synthase		c, g	
A-element	CAAAAAAA	*ZmSSI*	Starch synthase	*Zea mays*	h, g, k	Li et al. 2021 Min et al. 2019
		*OsSSI*		*Oryza sativa*	d, i	
	AANA	*AtPGR*	Plasma membrane Glc-regulated	*Arabidopsis*	c, d, e, i	
RY-element SUC/ROS-3 SUC/ROS-4 SUC/ROS-5 SUC/ROS-8 SUC/ROS-10	CATGC CATGCCTC CAGGCATG^+^ CATGCACC CATGCGGG CCTGCATG^+^			*Arabidopsis*	m m m m m	Geisler et al. 2006
GCGGG						Geisler et al. 2006 Li et al. 2006
SUC/ROS-8	CATGCGGG				m	
BS1EGCCR	AGCGGG		Carbohydrate metabolism enzymes		m	
I-box related						Li et al. 2006
IBOXCORENT	GATAAGR		Trehalose synthesis		m	
IBOXCORE	GATAA		Abiotic stress		m	
IBOX	GATAAG				m	
MYBST1	GGATA		Trehalose synthesis, abiotic stress and amino acid degradation		m	
AMYBOX2	TATCCAT^+^					
MYB26PS	GTTAGGTT		Carbohydrate metabolism and S uptake		m	Li et al. 2006

a, promoter deletion analysis; b, DNase footprinting; c, electrophoretic mobility shift assay; d, sequence; e, transactivation assay; f, mutation on the element; g, transient assay; h, one-hybrid system; i, mutant line (transgenics); j, SNP assay; k, DAPseq; l, ChIP; m, bioinformatic analysis; ^+^, indicates that the corresponding sequence is in the complementary strand (5′–3′); underline sequence are the ones that are shared in the group

**Fig. 2:**
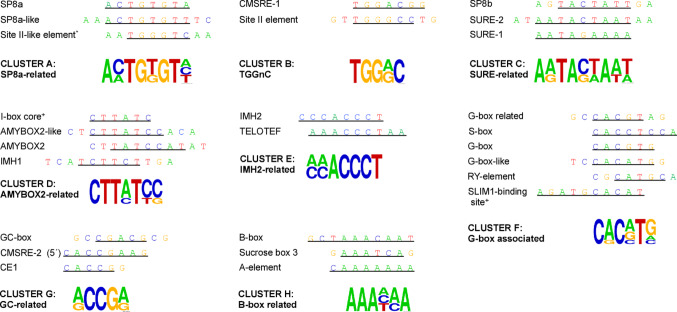
Clustering of sugar-responsive *cis*-regulatory elements (CREs). The CREs were grouped into clusters (A to H) based on sequence similarity, defined as the conservation of at least four-nucleotides shared by at least two elements within a cluster. The degree of sequence conservation within each cluster is visually represented as a sequence motif logo, generated with the WebLogo tool (https://weblogo.berkeley.edu). Underlined sequences represent the described in the literature as involved in sugar responsiveness CREs. ^+^, indicates that the corresponding sequence is located on the complementary strand

Sugars regulate a wide range of genes involved in diverse biological processes by modulating their transcription through TF-CRE interactions, leading to gene activation or repression (Koch 1996; Sheen et al. 1999; Price et al. 2004; Rolland et al. 2006; Biłas et al. 2016). Promoter analysis across different plant species have facilitated the identification of multiple CREs (Graham et al. 1994; Lu et al. 1998; Rolland et al. 2006), responsive to sugar availability or deprivation (Table 1). Based on previous reports (Sun et al. 2003; Sakr et al. 2018), an update comparison of sugar-responsive CREs is shown in Fig. 2. These clusters represent the seven recognized originally, SP8 (Cluster A, SP8a-related), CMSRE-1 (Cluster B, TGGnnn), SURE (Cluster C, SURE-related), TA-box (Cluster D, AMYBOX2-related), G-box (Cluster F, G-box associated), GC-box (Cluster G, GC-related) and B-box (Cluster H, B-box related), and includes the new one IMH2-related (Cluster E). The conserved sequence could suggest potential recognition by specific TF families and provides a comparative framework for understanding sugar-responsive elements across species and identifying potentially key consensus core sequences in sugar-mediated transcriptional regulation.

Historically, identification of sugar-responsive CREs began with the assessment of a few Suc-regulated genes encoding storage proteins in sweet potato (*Ipomoea batatas*), like sporamin and β-amylase (*β-Amy*) (Hattori et al. 1990; Nakamura et al. 1991); as well as patatin-1 in potato (*Solanum tuberosum*) (Grierson et al. 1994). Promoter deletion analysis revealed that multiple sugar-responsive CREs are present in the regulatory regions of these Suc-regulated genes. For instance, within the sporamin gene promoter, the SP8a (*ACTGTGTA*), CMSRE-1 (Carbohydrate metabolic signal-response element-1, *TGGACGG*), and CMSRE-2 (*AGACACCGTAAGTGTTCCA*) CREs were identified. Similarly, the β-amylase gene promoter harbored SP8a, SP8b (*TACTATT*) and CMSRE-1 (Table 1) (Hattori et al. 1990; Ishiguro and Nakamura 1992; Maeo et al. 2001; Morikami et al. 2005). Some of these sugar-responsive CREs were also found in promoters of storage-related genes in other plant species. For instance, in *Arabidopsis* the sugar-induced gene *β-Amy* contains CMSRE-1 (Mita et al. 1995), whereas in barley (*Hordeum vulgare*) *isoamylase1* (*iso1*) gene contains an SP8a-like element (*ACTGTGTT*) (Table 1) (Sun et al. 1999). Notably, SP8a and SP8a-like, differ by a single nucleotide, suggesting *ACTGTGT* as the consensus core sequence (Fig. 2, Cluster A: SP8a-related).

While much of the early work focused on storage-related genes, sugar-responsive CREs have also been identified in genes associated with other physiological processes, such as those found in the promoters of respiratory chain genes in *Arabidopsis* (Curi et al. 2003). For example, the *ATCATT* sequence mediates the sugar-induced expression of *COX5b-1* (Table 1). In contrast, the expression of *COX5b-2* is regulated by two related elements: the Site II element (*TGGGCC*) and the Site II-like element (*TGGGTC*) (Table 1) (Welchen et al. 2002; Comelli et al. 2009; Comelli and Gonzalez 2009), whereas the expression of *Cytc-2* depends on two Site II elements (Welchen et al. 2009). Although Site II elements have been found in other respiratory chain genes, its role in sugar response remains unclear, as these elements contribute to basal gene expression rather than directly mediating sugar responsiveness (Welchen and Gonzalez 2005; Mufarrege et al. 2009). Interestingly, sequence comparisons reveal that the Site II-like element shares five-nucleotides with SP8a, suggesting a conserved core represented by the sequence *A*(*C/A*)*TG*(*T/G*)*GT* (Fig. 2, Cluster A: SP8a-related). Furthermore, the Site II-like element shares a four-nucleotides core (*TGG*(*A/G*)*C*) with the CMSRE-1 element (Fig. 2, Cluster B: TGGnnC), which suggest a possible functional relationship between these elements in sugar-mediated gene regulation. Notably, the 3’ end of the Site II element includes the *GCCT* motif (Table 1), which was shown to mediate the Glc-induced repression of *PLASMA MEMBRANE GLUCOSE-RESPONSIVE REGULATOR* (*AtPGR*) gene (Chung et al. 2016).

In addition to these findings in respiratory genes, earlier studies comparing the sugar regulatory regions of tuber storage proteins, proteinase inhibitor II (*PI-II*), sporamin, and patatin-1 genes of potato, identified a conserved AT-rich sequence (Kim et al. 1991; Morikami et al. 2005). A more detailed analysis of patatin-1 regulatory region revealed three Suc-induction elements: a B-box (*GCTAAACAAT*), and two Sucrose responsive elements (SURE-1, *AATAGAAAA*, and SURE-2, *AATACTAAT*) (Table 1) (Grierson et al. 1994). Although the B-box element is present in other patatin genes, its role in sugar response remains unverified (Grierson et al. 1994). SURE-1, on the other hand, have been found to control gene expression of hexose transporter genes by the presence of sugars in other plant species, such as peach (*Prunus persica*) and grape (*Vitis vinifera*) (Table 1) (Atanassova et al. 2003; Cakir et al. 2003; Jiaxing et al. 2018); whereas similar sequences (SURE-a, *ATGGAAAA*; and -c, *AAAGAAAA*) regulate *iso1* gene in barley (Sun et al. 2003). Remarkably, a *TACTAA* sequence, resembling SURE-2 element, induces the expression of three genes of the cytochrome c oxidase subunit (*AtCOX6b*) in *Arabidopsis* (Table 1) (Mufarrege et al. 2009). Although Grierson and collaborators (1994) previously suggested homology between SUREs and SP8 elements, our alignment analysis confirms a conserved similarity between SURE and SP8b, particularly when two additional 5′ upstream bases are included. These elements possess a common core sequence *A*(*A/G*)*TA*(*C/G)*(*T/A*)*A*, which is characterized as a four-nucleotide core. The fact that they share this core suggests they belong to a cluster of related elements, designated as Cluster C (SURE-related) in Fig. 2.

Another mechanism by which sugar signaling regulates gene expression is derepression, especially under sugar starvation conditions. In this model, certain genes are actively repressed when sugars are abundant and become activated upon sugar depletion. A well-characterized example is the rice (*Oryza sativa*) α-amylase gene (*αAmy3*), which is induced in the absence of sugar (Huang et al. 1990). Promoter analysis identified three essential CREs required for this response: the AMYBOX2 (*TATCCAT*), the GC-box (*CGACG*), and a G-box related element (*CACGT*) (Table 1) (Hwang et al. 1998; Lu et al. 1998; Toyofuku et al. 1998). Later studies revealed that under sugar-deprived conditions, the TF OsMYBS1 binds to AMYBOX2 to activate *αAmy3*, whereas under sugar-rich condition, OsMYBS3 displaces OsMYBS1 at the same site to repress expression and maintain the gene in an off state (Lu et al. 2002). A similar regulatory mechanism has been proposed for the *ASPARAGINE SYNTHETASE* (*AS*) in asparagus (*Asparagus officinalis*), which is also induced by Suc deprivation. Its regulatory region contains both AMYBOX2 and AMYBOX2-like (*CTTATCCA*) elements (Table 1) (Winichayakul et al. 2004; Geisler et al. 2006). Comparative analyses of *AS* promoters in *Arabidopsis* (Lam et al. 1994), maize (*Zea mays*) (Chevalier et al. 1996), and bean (*Phaseolus vulgaris*) (Osuna et al. 2001), revealed a conserved AMYBOX2 element, reinforcing its conserved role in transcriptional regulation under sugar-starvation or as a repressive element in the presence of sugar.

Alignment of AMYBOX2 variants reveals a shared core motif, *TATCCA* (Fig. 2, Cluster D: AMYBOX2-related). Moreover, when considering two extra upstream bases, AMYBOX2 resembles the IMH1 element (*TCTTCT*) (Fig. 2, Cluster D: AMYBOX2-related). IMH1 along with IMH2 (*CCCA(C/A)CCT*), mediate sugar repression of the glyoxylate cycle genes *ISOCITRATE LYASE* (*ICL*) and *MALATE SYNTHASE* (*MS*) in cucumber (*Cucumis sativus*) (Graham et al. 1994; Reynolds and Smith 1995; Sarah et al. 1996; De Bellis et al. 1997). Interestingly, AMYBOX2, its variant AMYBOX2-like, and IMH1 share a consensus core sequence, *CTT(A/C)T(C/T*), which also overlaps with the complementary sequence of the I-box *GATAAG* core (Fig. 2, Cluster D: AMYBOX2-related), a CRE associated with light-regulated genes, and emerging as a sugar-responsive element. The I-box was identified as one of the top classifier elements of Glc-repressed genes in a machine learning study using Relevance Vector Machine (RVM) (see later section) (Li et al. 2006; López-Ochoa et al. 2007). These AMYBOX2-related elements may represent functional variants involved in sugar-mediated transcriptional repression.

In addition, the TELOTEF element (*AAACCCT*) (Table 1), identified together with the I-box and experimentally validated as sugar-responsive, shows strong sequence similarity to IMH2, sharing the core motif *ACCCT* (Fig. 2, Cluster E: IMH2-related). This similarity reinforces its potential role in sugar-responsive gene regulation.

Photosynthetic genes are also subject to sugar-mediated repression. For example, the *rbcS2* gene, encoding the small subunit of ribulose 1,5-bisphosphate carboxylase in bean, is repressed by Suc. The sugar regulatory region of this gene contains two E-box (*CANNTG*) variants, namely a perfect G-box (*CACGTG*), and a G-box-like sequence (*CACATG*), with an S-box (*CACCTCCA*) located between them (Table 1). Since the S-box was also found in the promoters of *rbcS* genes in other plant species, it has been proposed as a key element mediating sugar repression (Urwin and Jenkins 1997; Acevedo-Hernández et al. 2005). The functional role of the E-box variants, particularly the G-box, which is common in plant promoters, depends on the promoter context, including the combination and spacing with other CREs.

A regulator of the anthocyanin biosynthesis pathway, *PRODUCTION OF ANTHOCYANIN PIGMENT1* (*PAP1*), is activated by SULFUR LIMITATION 1 (SLIM1). SLIM1 binds to the *AGATGCACAT* sequence on the *PAP1* promoter upon the presence of Glc (Table 1) (Teng et al. 2005; Wawrzyńska et al. 2022). Notably, the last five nucleotides at the 3′ end of the *AGATGCACAT* sequence resemble the G-box-like element (*CACATG*). Given this clue, three G-box-associated elements (E-box variants), along with the S-box, and the *AGATGCACAT* sequence, were compared by sequence alignment. This analysis showed that these elements share the consensus *CAC(A/C/G*)*T* sequence (Fig. 2, Cluster F: G-box associated), suggesting a common evolutionary origin. Interestingly, the G-box-like shares a four-nucleotide with the RY element (*CATGCA*), best known for its role in seed maturation, abscisic acid (ABA) signaling, and sugar-mediated repression (see in later section) (Tsukagoshi et al. 2005). Together, these findings support the idea that G-box-associated elements, found in many plant genes responsive to multiple signaling pathways, act in combination with surrounding CREs to diversity transcriptional regulation in plants (Menkens et al. 1995; Ezer et al. 2017; Karr et al. 2022).

On the other hand, the *CACCG* motif was identified by incubating recombinant ZmABI4, a TF involved in the regulation of ABA and sugar responses (see in later section), with a library of random double-stranded DNA. The resulting protein-DNA complexes were separated via gel electrophoresis, and the shifted DNA was purified, amplified by PCR, and sequenced to identify the CRE for ZmABI4 binding (Niu et al. 2002). From this, the identified CRE was the Coupling Element (namely CE1; *CACCG*), which is also required for sugar induced expression of *ALCOHOL DEHYDROGENASE1* (*ADH1*), *PLASTOCYANIN* (*PC*), ADP-glucose pyrophosphorylase (*APL3*), starch branching enzyme (*SBE2.2*), *ABI4*, and *ABI5* (Table 1) (Niu et al. 2002; Arroyo et al. 2003; Bossi et al. 2009). Interestingly, the 5*’* end of the CMSRE-2 element harbors an identical CE1 motif and at the same time, it shares the (*A/G*)*CCG*(*A/G*) core with the GC-box (Fig. 2, Cluster G: GC-related).

Deletion analysis revealed that a second element near SURE-1, the Sucrose box 3 (S3S1, *AAATCA*), is involved in controlling the expression of *VvHT1* in grape (Atanassova et al. 2003; Cakir et al. 2003). Such element was also found in the *PpHT* promoter (Jiaxing et al. 2018), and is characterized by its A-rich sequence, similar to the B-box in *patatin-1* (Grierson et al. 1994) and the *CAAAAAAA* sequence (A-element) of the starch synthase enzyme gene in maize, and rice (Table 1). The A-element is the ZmPLATZ2 binding site, identified through DNA affinity purification sequencing (DAP-seq), and plays a role in promoting maize and rice starch synthase gene expression in response to Glc (Li et al. 2021). Notably, until now is the only sugar CRE identified through DAP-seq, a technique that uses a tagged TF immobilized on a matrix to screen a genomic DNA library (Table 2) (O’Malley et al. 2016; Bartlett et al. 2017). Sequence alignment showed similarities among these *cis*-elements, particularly in the region containing adenines (Fig. 2, Cluster H: B-box related).

**Table 2:** NGS techniques for identifying DNA specific regulatory regions containing CREs

Method	Principle	Advantages	Limitations	References
*Mapping open chromatin regions*				
ATAC-seq	Unfixed isolated nuclei are exposed to the Tn5 transposase, which simultaneously cleaves accessible chromatin regions and inserts adapter sequences. The adapters allow the construction of a library for next-generation sequencing	The protocol is fast and simple, requiring only a minimal number of cells. It has also been adapted for single-cell applications, enabling to analyze chromatin accessibility at the individual cell level	The Tn5 transposase might bind and cleave TF binding regions. Mitochondrial DNA contamination affects accuracy	Tsompana and Buck 2014 Sun et al. 2019
MNase-seq	Isolated crosslinked nuclei are treated with endo-/exonuclease MNase to digest accessible DNA. Reverse crosslinking and size selection of the DNA fragments is then performed to generate a library for sequencing. It works well for footprinting	DNA fragments remaining after MNase digestion represent protein-bound footprinting signals from both TFs and nucleosomes. Their analysis provides detailed insight into DNA accessibility and the interactions between nucleosomes and regulatory elements	The information obtained corresponds to the binding site of nucleosomes and TF. The reproducibility is low	Tsompana and Buck 2014 Zhao et al. 2020
DNase-seq	Unfixed isolated nuclei are treated with DNase that cleaves accessible chromatin. Short size DNA fragments are selected and ligated to a linker to generate a library for sequencing. It is used for footprinting	A reliable and robust method for detecting active regulatory regions, including promoters, enhancers and insulators, where regulatory factors interact with DNA. It is useful for comparing binding profiles across different conditions, providing valuable insights into gene regulation	Laborious enzyme titrations. The enzyme might cut the TFs binding sites	Tsompana and Buck 2014 Sullivan et al. 2015
FAIRE-seq	Chromatin crosslinked with formaldehyde and sheared by sonication. Then, a phenol chloroform extraction separates the nucleosomes and accessible DNA fragments. The accessible DNA is labeled and used to generate a sequencing library	It eliminates the need for single-cell suspensions or nuclear isolation, reducing complexity and increasing reproducibility. By avoiding enzymatic digestion, it minimizes sequence biases, leading to more consistent and reliable results	The background is high compared to other open chromatin assays what makes difficult the data analysis	Geresi et al. 2007 Murtha et al. 2015 Tsompana and Buck 2014
*TF binding site assays*				
ChIP-seq	The TF-DNA interaction is crosslinked and the chromatin is fragmented by sonication. The TF-DNA complex is immunoprecipitated with a specific antibody. The crosslinking is reversed, the DNA is precipitated and sequenced	It offers broad applicability, extensive coverage and a wide dynamic range, making ideal for TF binding studies using high-quality antibodies. With higher resolution and less noise, its sensitivity and specificity improve motif discovery and target identification	Quality of the antibody is crucial. The binding does not necessarily indicate a functional interaction	Park 2009 Ko and Brandizzi 2020
CUT&RUN-seq	The cells are sequentially incubated with an antibody against the TF and a Protein A fused to MNase that can bind to the antibody. MNase is activated to cleave the DNA around the binding site. The DNA fragments are released and diffused into the supernatant to be purified and sequence	It requires low input, making it ideal for small samples. It offers higher resolution than ChIP-seq through targeted digestion, reducing background noise while enhancing specificity. With fewer steps and lower sequencing depth, it efficiently maps TF binding and histone modifications	Over-digestion of the DNA by MNase	Kaya-Okur et al. 2020 Wang et al. 2023
CUT&Tag-seq	Replaced MNase with the Tn5 transposase. DNA cleavage and adapter ligation occurred simultaneously. Extraction of the DNA, generation of a library and sequencing	It requires less input than CUT&RUN, ideal for single-cell and limited samples. It offers high resolution with reduced DNA loss, low background noise and a streamlined without harsh processing. Suitable for native cells, it effectively profiles histones, TFs and cofactors	The tagmentation reduces occupancy of TFs in unfixed cells	Kaya-Okur et al. 2020
DAP-seq	Short fragments of genomic DNA are attached to adapters to generate a library. The TF fused to the Halo affinity tag is expressed in vitro and bound to magnetic beads. The HaloTag-TF is incubated with the DNA library. Unbound DNA fragments are washed away and the bound DNA is eluted, size-selected, and sequenced	It systematically maps TF binding to naked DNA, making it ideal for high-throughput motif discovery. Using small amounts of genomic DNA and affinity-tagged proteins, it provides detailed insight into DNA-binding proteins and their target sites	Low success rate (~ 30%)	O’Malley et al. 2016 Bartlet et al. 2017

In watermelon (*Citrullus lanatus*), the *Tonoplast Sugar Transporter 2* (*ClTST2*) gene, encoding a vacuolar sugar transporter, is regulated by SUGAR SIGNALING IN WATERMELON1 (SUSIWM1), a SUGAR SIGNALING IN BARLEY2-like (SUSIBA2-like) TF that binds to a W-box found in its promoter (Ren et al. 2018). A similar regulatory feature is observed in melon (*Cucumis melo*), where the *CmTST2* gene, which is induced by sugar accumulation, also contains a W-box in its promoter (Cheng et al. 2018). The W-box has likewise been identified in the promoters of *HpINV2* and *HpSuSy1*, two genes involved in Suc metabolism in pitaya (*Hylocereus polyrhizus*), regulated by the sugar-responsive TF HpWRKY3 (Wei et al. 2019). Collectively, these findings support the notion that the W-box is an emerging CRE in sugar signaling. Although it lacks sequence similarity with other known sugar-responsive CREs, its presence in the promoters of sugar-induced genes across species it may represent an additional regulatory layer involved in sugar signaling.

Additionally, the *GAGA* motif has been implicated in sugar-regulated gene expression, as it is associated with the activation of *AtPGR* in response to Glc through the TF bHLH34 (Min et al. 2017). The *GAGA* motif is embedded within the Suc-6 (*GAANGAGANGA*) element (Table 1), which was identified through bioinformatic analysis as a potential sugar-responsive element (see later section).

## Genome-wide identification of sugar-responsive CREs: integrating sequencing technologies and bioinformatics

Genome-wide approaches have significantly advanced the identification of regulatory elements involved in gene expression. Although all these methods rely on bioinformatic data analysis, they differ in how functional information is experimentally obtained. The three main strategies include (i) analyzing sugar-regulated genes to identify overrepresented CREs, (ii) mapping open chromatin regions, and (iii) performing comprehensive TF-binding assays to determine TF binding sites. These approaches utilize NGS technologies, providing valuable information for dissecting gene regulation. Early studies employed the first approach and relied mainly on microarray data before the widespread adoption of NGS (Li et al. 2006; Chow et al. 2018; Galli et al. 2020; Schmitz et al. 2022).

*Analyzing sugar-regulated genes to identify CREs*: Bioinformatic analyses help identify overrepresented CREs within gene sets expressed under specific conditions, such as sugar treatments. These analyses rely on developing algorithms that correlate transcriptomic expression profiles with promoter sequence data, enabling the inference of functional regulatory networks (Geisler et al. 2006; Li et al. 2006). Whereas bioinformatics can predict a large number of putative CREs, only a fraction can be experimentally validated and confirmed to mediate sugar-responsive gene expression. Another limitation of this approach is the inherent shorth length of CREs, which leads to their widespread occurrence throughout the genome, making it difficult to distinguish functional elements from background sequences (Geisler et al. 2006; Li et al. 2006; Ho and Geisler 2019). Despite these limitations, validated sugar-responsive CREs, identified through this approach, can often be traced across plant species through sequence comparison and conservation analyses (Galli et al. 2020).

Geisler et al. (2006) investigated the distribution of CREs across the *Arabidopsis* genome by correlating their native occurrence in regulatory regions with microarray data from seedlings grown on Suc and light, darkness, or at high Suc concentration. This study identified twelve CREs, SUC/ROS-1 to SUC/ROS-10, Suc-6, and Sucrose repressor, of which SUC/ROS-5, and SUC/ROS-6 were experimentally validated as Suc-responsive (Geisler et al. 2006) (Table 1). Whereas many of the remaining elements have not yet been functionally characterized, several exhibit notable sequence similarity to previously described sugar-responsive motifs. For instance, SUC/ROS-1, and -2 have similarities to IMH2, whereas SUC/ROS-3, -4, -5, -8 and -10 share a conserved RY element (Table 1). The confirmed sugar responsiveness of SUC/ROS-5 supports the functional relevance of this RY element. SUC/ROS-6 shares the core sequence *TGGA*(*C*/*T*)*G* with CMSRE-1, and SUC/ROS-7 and SUC/ROS-9 contain *GGCAGG*, which partially overlaps with CMSRE-1 by four nucleotides, differing only in the central bases. Additionally, Suc-6 shows sequence similarity to both IMH1 and *GAGA* motif, whereas the Sucrose repressor contains AMYBOX2 and AMYBOX2-like sequences (Table 1). These findings suggest that many of these elements may represent functional variants of known sugar-responsive CREs, although further experimental validation is needed.

Similarly, Li et al. (2006) employed a Bayesian analysis using RVM, a machine learning approach, to classify plant promoters based on microarray data from *Arabidopsis* seedlings treated with 3% Glc. This analysis led to the identification of around 14 putative CREs, most of which share similarities with known sugar-responsive motifs (Table 1) (Li et al. 2006). For example, QARBNEXTA and LRENPCABE resemble the G-box, and EVENINGAT harbors a sequence similar to the Sucrose box 3 (Table 1). E2FBNTRNR includes the *GGCA* core motif, characteristic of certain SUC/ROS elements (Table 1), and BS1EGCCR is highly similar to SUC/ROS-8 with the sequence *GCGGG* (Table 1). Moreover, four CREs (IBOXCORENT, IBOXCORE, IBOX, and MYBST1) clustered together due to the presence of the I-box (Table 1), and share a four-nucleotide sequence similarity with the AMYBOX2 element (Li et al. 2006). DRECRTCOREAT is similar to the GC-box sequence, and GARE2OSREP1 shares a four-nucleotide core with the SP8b element (Table 1), suggesting a derived variant of SP8b. Among the motifs identified, TELOTEF was experimentally validated as sugar-responsive and shows sequence similarity to IMH2 and the complementary strand of AMMORESIIUDCRNIA1 (Li et al. 2006). Lastly, MYB26PS remains with no clear homology, thus representing a candidate for future validation.

*Chromatin accessibility mapping and TF-binding assays*: In contrast to the previously described method, these approaches provide direct experimental evidence of regulatory regions. Chromatin accessibility mapping identifies open chromatin regions (OCRs) associated with active regulatory elements. The accessibility of these regions can be assessed using techniques that involve isolating the plant nuclear genome, followed by a treatment with endonucleases, separating the resulting DNA fragments (nucleosome-free or nucleosome-protected), and subsequently identifying and quantifying these fragments using NGS platforms (Tsompana and Buck 2014; Galli et al. 2020; Ko and Brandizzi 2020; Schmitz et al. 2022). Currently, four strategies to map OCRs are: Assay for Transposase Accessible Chromatin (ATAC)-seq (Sun et al. 2019), Micrococcal Nuclease hypersensitivity (MNase)-seq (Zhao et al. 2020), DNase hypersensitivity (DNase)-seq (Sullivan et al. 2015), and Formaldehyde-Assisted Isolation of Regulatory Elements (FAIRE)-seq (Giresi et al. 2007; Murtha et al. 2015). Despite challenges in ensuring reproducibility across replicates, these methods are powerful for studying the relationship between DNA accessibility, gene expression and downstream developmental outcomes (Tsompana and Buck 2014; Ko and Brandizzi 2020) (see details in Table 2).

For mapping TF DNA-binding regions, the predominant technique is chromatin immunoprecipitation (ChIP). The ChIP protocol involves crosslinking TF-DNA complexes, chromatin fragmentation, isolation of TF-DNA complexes using antibodies, and DNA precipitation followed by high-through input sequencing (Park 2009; Swift and Coruzzi 2017). Modified versions of this protocol have led to: Cleavage Under Targets and Release Using Nuclease (CUT&RUN)-seq, as well as CUT and Tagmentation (CUT&Tag)-seq. In both approaches, an antibody targeting the TF is linked to a DNA-cleaving enzyme, improving resolution and efficiency (Kaya-Okur et al. 2020; Wang et al. 2023). Each technique has its limitations, including DNA yield, but together they offer a comprehensive approach to identifying regulatory elements (Table 2) (Park 2009; Swift and Coruzzi 2017).

By integrating chromatin accessibility data with TF-binding profiles, the identification of functional CREs can be refined, enabling the establishment of direct regulatory connections between TFs and target genes. This integrative approach is particularly relevant to sugar response, as these techniques have largely not been applied in this context. This integrated framework will enhance our understanding of sugar-responsive gene regulation. Computationally predicted CREs serve as a base resource for investigating sugar-regulated gene expression and identify potential candidates for functional validation. Furthermore, sequence similarities across these CREs suggest a conserved regulatory mechanism underlying sugar responses across gene networks.

The CREs listed in Table 1, along with their clustering patterns (Fig. 2), provide a starting point to understand sugar-mediated response in an integral manner. Clusters of related CREs may indicate shared regulatory mechanisms that govern expression of genes involved in sugar transport, metabolism, respiration, storage, and signaling. Understanding these regulatory networks will be essential for engineering crops with improved traits through precise gene expression modulation.

## Transcription factor families and their roles in sugar signaling

To control gene expression, CREs act on the same DNA molecule, whereas *trans-*regulatory elements, as TFs, act on other genomic regions. The interplay between these elements dictates timing, location, and rate of gene transcription. Since some CREs are present in multiple genes, the presence and concentration of specific TFs are determinants for driving gene expression in particular tissues and conditions (Carey 1998; Chung et al. 2016; Hoang et al. 2017). Mostly, TFs fulfill their function by binding to specific CREs through their DNA-binding domains, while interacting with the PIC via their transcription effector domains. These interactions lead to either activation or repression of transcription. Interestingly, a single TF may act as an activator for one gene and a repressor for another, depending on the recruitment of co-activators or co-repressors and the regulatory context (Fuda et al. 2009; Bemer et al. 2017; Hoang et al. 2017; Hummel et al. 2023). In addition, TFs often form homodimers or heterodimers, which expands their DNA-binding specificity and functional versatility. Based on their DNA-binding domains, TFs are grouped into various families; each comprising multiple members that regulate diverse biological processes, such as growth, development and stress responses (Seo et al. 2015; Strader et al. 2022).

Several TF families have been implicated in sugar responses across different plant species, often through interactions with specific CREs (Table 3). Based on their affinity to bind specific CREs, the one-hybrid system has been widely used to identify sugar-responsive TFs by fusing a sugar-responsive CREs (or a fragment harboring them) to a reporter gene that serves as bait to screen cDNA libraries derived from sugar-treated or untreated tissues. This approach allows the identification of those TFs that specifically recognize and bind to these regulatory sequences of interest. Once a TF is identified as a true regulator in one plant species, cross-species sequence comparison allows the identification of putative ortholog TFs, thereby inferring their role in other plants (Yang 1998; Dominguez et al. 2013; Chung et al. 2016; Chen et al. 2017; Jiaxing et al. 2018; Galli et al. 2020).

**Table 3:** Members of transcription factor families associated with the regulation of sugar-responsive genes in diverse plant species

Family	TF	Species	Target	Binding CRE	Method	References
ASR	VvMSA	*Vitis vinifera*	*VvHT1*	Sucrose box 3 and SURE-1	A	Cakir et al. 2003
	PpASR	*Prunus persica*	*PpHT*	Sucrose box 3	B, C	Jiaxing et al. 2018
	FaASR	*Fragaria x annanassa*	*FaHT1*, *FaHT2* and *FaHT3*	Sucrose box 3	B, C	Jia et al. 2016
	SlASR1	*Solanum lycopersicum*	*SlHT1*	Sucrose box 3	B, C	Jia et al. 2016
	SlASR1/ci21A	*Solanum tuberosum*	*HvHT* and *StHT2*		D, E	Frankel et al. 2007; Shkolnik and Bar-Zvi 2008; Dominguez et al. 2013
		*Nicotiana tabacum*	*NtHT1*, *NtSUT2*, *HXK1* and *SnRK1.1*		D, E	
		*Arabidopsis*	*ABI4*, *ABI5, EM6* and *ADH1*		D, G	
	OsASR1	*Oryza sativa*	*MST*, *SUT*, *CIN2* and* CIN5*		D	Joo and Song 2015
	ZmASR1*	*Zea mays*				Virlouvet et al. 2011
	ZmASR5*					
	mAsr1*	*Musa*				Henry et al. 2011
	mAsr3*					
bZIP	bZIP1	*Arabidopsis*	*ASN1* and *ProDH*	G-box and ACT motif	D, F, G, H, I	Kang et al. 2010
	bZIP3		Leaf development genes		D	Sanagi et al. 2018
	bZIP11/ATB2		*ProDH2*, *ASN1*, *TRE1*, *TPP5*, *TPP6* and carbon metabolism genes	G-box and related	C, D, H, J, K	Hanson et al. 2008; Ma et al. 2011
	bZIP53		*ASN1* and *ProDH*	G-box and ACT motif	D, F, G, I	
	bZIP63		*ASN1*, *SEX1* and *BAM9*	G-box	F, G, H, K	Matiolli et al. 2011; Viana et al. 2021
	SlbZIP1	*Solanum lycopersicum*	*ProDH* and *ASN*		B, C, D	Sagor et al. 2016
	SlbZIP2				B, C, D	
	ClbZIP1	*Citrullus lanatus*	*ClPHT4;2*		A, I	Zhang et al. 2017
	ClbZIP2				A, I	
	TBZ18	*Nicotiana benthamiana*	*ProDH* and *ASN*		B, D	Thalor et al. 2012
MYB	OsMYBS1	*Oryza sativa*	*αAmy3*	AMYBOX2	A, C, L	Lu et al. 2002
	OsMYBS2				A, C, L	
	OsMYBS3				A, C, L	
	MYBS1	*Arabidopsis*	*HXK1*, *APL3*, *CHS*, *CAB1* and *ASN*	AMYBOX2	B, F	Chen et al. 2017
	MYBS2				B, F	
	CSA	*Oryza sativa*	*MST8*, *Amylase 3A* and *Os3BGlu6*	CCAAT box	C, F, G	Sun et al. 2021
WRKY	SPF1	*Ipomoea batatas*	*gSPO-A1*, *gSPO-B1* and *gβ-Amy*	SP8a and SP8b	A, L	Ishiguro and Nakamura 1994
	SUSIBA2	*Hordeum vulgare*	*iso1*	SURE and W box	B, L	Sun et al. 2003
	SUSIWM1	*Citrullus lanatus*	*ClTST2*	W-box	A, C, L	Ren et al. 2018
	HpWRKY3	*Hylocereus polyrhizus*	*HpINV2* and *HpSuSy1*	W-box	A, I, L	Wei et al. 2019
bHLH	bHLH34	*Arabidopsis*	*AtPGR*	GAGA and E-box	A, C, D, E, L	Min et al. 2017; 2019
	bHLH104			AANA and E-box	B, D, G, L	
	MdbHLH3	*Malus domestica*	*MdMYB1*, *MdANS*, *MdUFGT*, *MdPFPβ,* anthocyanin biosynthesis and regulatory MYB genes	G-box	D, L, M, N, O	Hu et al. 2016; Yu et al. 2022
AP2	WRI1/ASML1	*Arabidopsis*	Glycolysis and triacylglycerol biosynthesis genes		I, P	Masaki et al. 2005b
	ABI4		*ABI4*, *ABI5*, *SBE2.2*, *RBCS, Lhcb* and seed maintainance genes	S-box, CE1 and -like	P	Acevedo-Hernandez et al. 2005; Bossi et al. 2009
	ZmABI4	*Zea mays*	*ADH1*	CE1 and -like	B, L	Niu et al. 2002
	ZmEREB156		*ZmSSIIIa*		A, I, O	Huang et al. 2016
B3 domain	HSI2/VAL1	*Arabidopsis*	Embryonic trait genes	RY element	F, H, P	Tsukagoshi et al. 2005; Susuki et al. 2007
	HSL1/VAL2				F, H, P	
EIN3/EIL	EIN3	*Arabidopsis*			D, F	Yanagisawa et al. 2003
	SLIM1		*PAP1* and anthocyanin biosynthesis genes	AGATGCACAT	B, C, F	Wawrzynska et al. 2022
CCT domain	ASML2	*Arabidopsis*	*β-Amy*, *ApL3* and *VSP2*		P	Masaki et al. 2005b
HD-Zip	ATHB13	*Arabidopsis*	*β-Amy, VSP* and development of coyledons and leaves genes		D	Hanson et al. 2001
PLATZ	ZmPLATZ2	*Zea mays*	*SSI*, *SIIa*, *WX* and other starch biosynthesis genes2	CAAAAAAA	A, D, I, R	Li et al. 2021
DUF domain	STK	*Solanum tuberosum*	*pat21*	B-box	L, Q	Zourelidou et al. 2002
	AtSTKL1	*Arabidopsis*	*AtPGR*	GCCT	A, D, E	Chung et al. 2016
	AtSTKL2				B, D, E	
	SlSTK	*Solanum lycopersicum*			B, D, F	Lu et al. 2023

A, yeast one-hybrid system; B, homolog; C, transactivation assay; D, overexpression; E, antisense; F, mutant; G, ChIP; H, microarray; I, transitory assay; J, algorithms; K, Gas Chromatography Time of Flight Mass Spectrometer; L, EMSA; M, co-immunoprecipitation; N, yeast two-hybrid system; O, RNAseq; P, mutant screening; Q, southwestern screening. *, Since their expression is regulated by sugars, it is speculated that they may also be involved in sugar signaling, similar to other TFs

Although the one-hybrid system is a powerful technique, it has certain limitations. As with most in vitro techniques, it does not consider the dynamic activity of TFs in vivo, including their binding kinetics, or reliance on other factors or cofactors within the native environment of a living cell (Swift and Coruzzi 2017). Despite these limitations the one-hybrid system remains a valuable tool for identifying TFs that interact with specific promoter segments or regulatory elements. An alternative strategy for uncovering sugar-related TFs involves the isolation of T-DNA and EMS mutants with altered germination or developmental responses to sugar exposure (Chen et al. 2017; Min et al. 2017; Wawrzyńska et al. 2022). These screenings have permitted the identification of mutants that, unlike wild-type plants which are unable to grow and become arrested at early developmental stages under high sugar concentrations, are able to overcome this developmental impediment. Therefore, these mutants are recognized as having a sugar hyposensitivity phenotype, such as the well-known *gin* (*glucose-insensitive*), *sis* (*sugar insensitive*), and *isi* (*impaired sugar induction*) mutants, among others. In counterpart, mutants that cannot grow or development under low sugar concentration, conditions under which wild-type plants develop normally, display sugar hypersensitive. Examples include *glo* (*glucose oversensitive*), *gss* (*glucose supersensitive*), and *hsr* (*high sugar-response*) mutants (Ramon et al. 2008). These genetic screenings have facilitated the identification of multiple TFs involved in sugar signaling pathways across different plant species. This section describes the TF families that have been functionally implicated in sugar signaling pathways, along with their corresponding CREs, if identified (Table 3).

### ASR (ABA-, stress-, and ripening-induced proteins) family

ASR proteins are typically around 13–25 kDa, characterized by their ABA/WDS (abscisic acid/water-deficit stress) amino acid domain (Gilad et al. 1997; Cakir et al. 2003). Some ASR members possess a zinc-dependent DNA-binding domain, enabling them to function as TFs, with the ability to form homo- and heterodimers. These TFs mediate responses to ABA, stress, and fruit development. ASR proteins are present in various plant species including rice, maize, beans, potatoes, peach and grape; however, in *Arabidopsis* ASR proteins are absent (Saumonneau et al. 2012). Whereas ABA is the main regulator of *ASR* gene expression, the sugar-induced expression of some *ASR* genes suggests their participation in sugar signaling (Iusem et al. 1993; Gilad et al. 1997; Saumonneau et al. 2012).

The maturation-, stress-, ABA-induced (VvMSA) protein, an ASR homolog in grape, was identified through one-hybrid approach using the sugar-responsive region of *VvHT1* gene (Cakir et al. 2003). VvMSA binds to both Sucrose box 3 and SURE-1 elements (Table 3). In addition, the analysis of grape cells, in which VvMSA was silenced by RNAi, showed alterations in the activities of specific glycolytic enzymes in response to sugar, suggesting the involvement of VvMSA in primary metabolism. Like *VvHT1*, *VvMSA* expression is induced by both sugars and ABA indicating that there is a mechanism of reinforcement to ensure a proper response (Cakir et al. 2003; Saumonneau et al. 2012; Parrilla et al. 2022). Homologs of VvMSA have been identified in peach (PpASR), strawberry (*Fragaria x ananassa*) (FaASR), and tomato (*Solanum lycopersicum*) (SlASR1), where these TFs mediate the induction of *HT* genes (Table 3). In strawberry and tomato, the suppression of *ASR* expression by RNAi resulted in delayed expression of ripening-related genes, and in the production of immature, hard, and green fruits; whereas *ASR* overexpression led to the up-regulation of genes associated with cell wall and anthocyanin metabolism, resulting in increased anthocyanin accumulation and softer fruits (Chen et al. 2011; Jia et al. 2016; Jiaxing et al. 2018).

Further studies involving the artificial manipulation of *SlAsr1* expression in various plant species revealed diverse phenotypic outcomes, impacting sugar metabolism, carbon allocation, and hormone levels (Frankel et al. 2007; Shkolnik and Bar-Zvi 2008; Dominguez et al. 2013). In transgenic potato plants, the antisense expression of *SlAsr1* (orthologue in potato, *ci21A*) resulted in reduced tuber weight, elevated Glc levels, and increased expression of an *Ht* (*Hexose transporter*) gene. In contrast, overexpression of *SlAsr1* led to a decrease in the number of tubers, reduced Glc concentration, and lower *StHT2* transcript levels (Frankel et al. 2007). On the other hand, *SlAsr1* RNAi expressing tobacco plants exhibited stunted growth, delayed flowering time, and increased Glc content, accompanied by reduce ABA levels in leaves. Additionally, sugar transporter genes (*NtHT1* and *NtSUT2*) were down-regulated, whereas expression of Glc signaling components (*HXK1* and *SnRK1.1*) was altered (Dominguez et al. 2013). Overexpression of *SlAsr1* in *Arabidopsis* caused ABA and Glc insensitivity in seeds, resembling the *abi4* (*abscisic insensitive4*) mutant phenotype (Shkolnik and Bar-Zvi 2008). Transcript levels of *ABI4* and its target genes (*ABI5*, *EM6*, *ADH1*, and *ZEP1*) were reduced upon Glc treatment, suggesting that SlASR1 is involved in sugar mediated gene regulation, likely through the CE1-like motif (Iusem et al. 1993; Shkolnik and Bar-Zvi 2008; Dominguez et al. 2013). These findings are surprising since ASR1 is absent in *Arabidopsis*; however, this suggests that an ASR1-like regulatory network does exist in *Arabidopsis*.

In monocots species such as rice, overexpression of *OsASR1* altered sugar levels in roots and enhanced sugar responses in the presence of ABA (Joo and Song 2015). In addition, transcript levels of sugar transporters and Suc metabolism-related genes (*MST*, *SUT*, *CIN2* and *CIN5*) were also affected. Notably, *OsASR1* overexpressing plants exhibited a hypersensitive root growth phenotype compared to the wild-type when exposed to 10% of Glc (Joo and Song 2015).

Given the likely correlation between *ASR*s expression and sugar-mediated responses, homologs of the ASR protein in maize (ZmASR1 and ZmASR5), and banana (*Musa x paradisiaca*) (mAsr1 and mAsr3) are also proposed to participate in sugar signaling. In fact, the expression of *ZmASR*s genes is induced by Glc, whereas *mAsr*s genes are induced by Suc instead (Henry et al. 2011; Virlouvet et al. 2011). Overall, these reports collectively highlight the putative role of ASR proteins in sugar signaling, influencing sugar metabolism, carbon partitioning, anthocyanin biosynthesis, cell wall metabolism, and fruit development. Genes regulated by ASRs and associated with the mentioned processes are expressed in diverse plant tissues, including fruits, tubers, and leaves (Frankel et al. 2007; Jia et al. 2016; Parrilla et al. 2022). However, whether ASRs regulate their targets directly or act through intermediates remain to be determined.

### bZIP (basic region leucine zipper) family

The bZIP domain of approximately 60–80 amino acids, contains a basic region responsible for DNA binding and a leucine zipper region that facilitates homo- or heterodimerization (Hong 2016). bZIP TFs regulate diverse biological processes, including light signaling, abiotic stress responses, pathogen defense, seed maturation, flower development, and nutrient and stress signaling (Seo et al. 2015; Ng et al. 2018). In *Arabidopsis*, the bZIP family is divided into 13 subfamilies, one of which is the S-group, characterized by a unique translation regulation mechanism termed Suc-induced repression of translation (SIRT). The SIRT mechanism involves the translation of an upstream open reading frame (ORF) located in the 5’UTR of genes associated with the Suc signaling pathway. Gene members of this subfamily, such as bZIP1, -2, -11, -44, and -53, are regulated through this mechanism (Wiese et al. 2004; Corrêa et al. 2008; Kang et al. 2010).

Transcriptomic analysis of plants overexpressing *bZIP11* revealed its impact on genes associated with amino acid and trehalose metabolism (Hanson et al. 2008). A co-expression assay corroborated some direct targets of bZIP11, such as *ASN1, ProDH2, TRE1, TPP5* and *TPP6*. Within the promoters of up-regulated genes by bZIP11, G-box related elements were identified as putative binding sites, whereas no consensus sequence was found for the down-regulated genes (Hanson et al. 2008; Ma et al. 2011). In fact, a mutation of the G-box within the promoters of *ASN1* and *ProDH2* demonstrated that this element is important for their expression. Additionally, a metabolic analysis showed that carbohydrate metabolite profiles were altered in transgenic plants overexpressing *bZIP11*. Such effect was enhanced in the presence of sugars (Glc, Suc, or trehalose), correlating with their growth inhibition phenotype (Hanson et al. 2008; Ma et al. 2011). Homologs of bZIP11 have been identified in tomato and tobacco, two homologs SlbZIP1 and SlbZIP2 and single counterpart TBZ18, respectively (Table 3). Similar to findings in *Arabidopsis*, these bZIPs also regulate *ASN* and *ProDH*, resulting in changes in the expression of genes involved in Suc synthesis (Thalor et al. 2012; Sagor et al. 2016).

In addition to bZIP11, the bZIP1, and bZIP53 TFs also regulate the expression of *ASN1* and *ProDH* in *Arabidopsis*, by binding to G-box and *ACT* (*ACTCAT*) motifs during sugar starvation (Dietrich et al. 2011) (Table 3). Although no phenotype in *bzip53* was observed, the *bzip1* mutant showed a higher percentage of seedlings with true leaves under sugar deprivation, whereas its overexpression produced the opposite effect compared to the wild-type, suggesting that bZIP1 is implicated in leaf development mediated by sugar. Furthermore, a microarray gene expression assay identified 516 mis-regulated genes (256 up- and 260 down-regulated) in the *bzip1* mutant, and 470 mis-regulated genes (221 up- and 249 down-regulated) in *bZIP1* overexpressor plants (Kang et al. 2010). In both cases, a high proportion of these genes corresponded to Glc-responsive genes. In addition, several bZIP1 interactors have been identified; some belonging to the S-group (bZIP11, -44, and -53), and others to the C-group (bZIP10, -25, and -63) (Kang et al. 2010; Dietrich et al. 2011). Another bZIP proposed to regulate sugar responses is bZIP3 because its overexpression resulted in abnormal cotyledons, exhibiting hyponastic bending and irregular surfaces when grown in 1% Suc (Sanagi et al. 2018) (Table 3).

In *Arabidopsis*, bZIP63, a direct target of SnRK1, mediates starch degradation and energy deficit responses (Viana et al. 2021). Accordingly, the *bzip63* mutant showed altered expression levels of *SEN1*, *DIN10*, *PWD*, *DPE2*, *ASN1*, *SEX1*, and *BAM9*; being the last three genes direct targets of bZIP63 (Matiolli et al. 2011; Viana et al. 2021). Additionally, the *bzip63* mutant has an accelerated starch degradation and reduced levels of carbon-related metabolites. Since starch degradation maintains sugar supply during nocturnal growth and is regulated by the circadian clock, the *bzip63* mutant exhibits a deficit in energy at nighttime. Therefore, bZIP63 links energy status and the circadian clock, optimizing carbon utilization throughout the day-night cycle (Baena-González et al. 2007; Viana et al. 2021) (Table 3). Beyond Arabidopsis, bZIP in other species also contribute to sugar signaling. For example, in watermelon, ABA and sugar accumulation regulate the expression of the plasma membrane Pi transporter gene (*ClPHT4;2*) during fruit development. Such regulation is through ClbZIP1 and ClbZIP2, where ClbZIP1 activates *ClPHT4;2*, whereas ClbZIP2 represses it (Zhang et al. 2017). Homologs of ClbZIP1 and ClbZIP2 in *Arabidopsis* are ABI5 and bZIP44, respectively; whose expression is also regulated by sugar (Zhang et al. 2017) (Table 3). Based on these findings and given that bZIP44 is regulated by SIRT, ClbZIP2 emerges as a potential candidate in sugar signaling in watermelon (Zhang et al. 2017). However, further studies are required to determine the specific roles of ClbZIP1 and -2 in this context. Finally, since most of the described *bZIPs* genes are also regulated by sugars, mainly downregulated, this suggests the existence of a regulatory loop between these TFs and sugar-derived responses.

### MYB family

MYB proteins constitute the largest TF family in plants (Hong 2016; Ng et al. 2018). The MYB domain is composed of up to four imperfect amino acid sequence repeats (R), each approximately 52 amino acids in length, which forms a helix-turn-helix structure that intercalates into the major groove of DNA (Hong 2016). Depending on the number of R repeats, MYB proteins are classified into subfamilies. MYB TFs regulate diverse plant-specific processes, including secondary metabolism, cell fate and identity regulation, and responses to biotic and abiotic stresses (Ng et al. 2018).

Rice OsMYBS1, OsMYBS2, and OsMYBS3 were identified through a one-hybrid system using as bait the AMYBOX2 motif of *αAmy3* (Lu et al. 2002) (Table 3). EMSA assays showed that these OsMYBS exhibited different binding affinities to AMYBOX2. On the other hand, co-transfection assays in barley aleurone cells treated with sugar demonstrated that OsMYBS1 and OsMYBS2 are strong and weak transcriptional activators, respectively, under sugar-starved conditions, whereas OsMYBS3 functions as a repressor during plant senescence (Lu et al. 2002). The weak activation by OsMYBS2 might be due to specific neighboring sequences required by interactors (Chen et al. 2002; Lu et al. 2002). Interestingly, the homologs of OsMYBS1 in *Arabidopsis*, MYBS1 and MYBS2 (Table 3), retain the ability to bind AMYBOX2 and regulate the expression of sugar-responsive genes (Chen et al. 2017). Independent mutations of these TFs in *Arabidopsis* exhibit a Glc-hypersensitivity and -hyposensitivity phenotypes, respectively; whereas the double mutant *mybs1 mybs2* restores the wild-type phenotype (Chen et al. 2017). Expression analysis revealed that the Glc up- and down-regulated genes showed opposite expression patterns in *mybs1* and *mybs2* mutants. In the former group, *HXK1*, *APL3*, *CAB*, and *CHS*, showed higher and lower levels of expression in *mybs1* and *mybs2*, respectively, compared to wild-type. In contrast, Glc down-regulated genes, such as *CAB1*, and *ASN* genes, showed a reduced expression in *mybs1* and an increased expression in *mybs2*, compared to the wild-type. Additionally, the expression of some genes involved in ABA biosynthesis and signaling were increased in *mybs1*, and decreased in *mybs2.* Such misregulation correlated with the phenotypes of *mybs1* and *mybs2*; exhibiting hypersensitivity and hyposensitivity to ABA, respectively. Altogether, these findings suggest that, although structurally related TFs may share a common binding CRE, they may have distinct mechanisms for regulating sugar-responsive genes, and even involved in different signaling pathways (Chen et al. 2017). Again, sugar and ABA seem to be interlinked in the regulation by MYBS.

Another MYB TF involved in sugar signaling is the Carbon Starved Anther (CSA) in rice (Table 3). CSA regulates early seed germination, anther sugar partitioning, pollen development and male fertility (Zhang et al. 2010). The *csa* mutant displays higher germination rate, altered expression of genes involved in carbohydrate and ABA metabolism, increased Glc levels due to elevated amylase activity, reduced ABA levels, and reduced sensitivity to exogenous Glc and ABA compared to the wild-type rice (Zhang et al. 2010; Sun et al. 2021). Direct targets of CSA include *Amylase 3A*, *Os3BGlu6* and *MST8*, encoding an α-amylase, a β-glucosidase, and a monosaccharide transporter of the anther vascular bundle, respectively (Zhang et al. 2010; Sun et al. 2021). Their promoters contain the putative binding site of CSA, a *CCAAT*-box that is similar to IMH2. Further evaluation revealed that CSA represses *Amylase 3A* and induces *Os3BGlu6* expression (Sun et al. 2021). Altogether, all evidence suggests that CSA balances Glc and hormone metabolism, serving as a signal to regulate gene expression in specific plant tissues (Zhang et al. 2010; Sun et al. 2021).

As observed for bZIPs, MYB TFs are also transcriptionally regulated by sugars, suggesting a possible regulatory feedback loop. For instance, *OsMYBS2* is Suc- and Glc-induced, whereas *OsMYBS1* and *OsMYBS3* are upregulated under sugar starvation. On the other hand, transcription of *MYBS1* and *MYBS2* in *Arabidopsis* is triggered by Glc; however, they have opposite functions in sugar signaling (Lu et al. 2002; Chen et al. 2017).

### WRKY family

The WRKYGQK motif within the 60 amino acid WRKY domain gives the name of this family. The structure of this domain comprises four β-sheet strands at the N-terminal and a zinc-finger-like motif at the C-terminal (Hong 2016). Although alternative binding sites have been identified, members of this family predominantly bind to the W-box sequence (Seo et al. 2015). WRKY TFs regulate diverse developmental and physiological processes, including embryogenesis, trichome development, anthocyanin biosynthesis, and responses to biotic and abiotic stress (Seo et al. 2015; Ng et al. 2018).

The SP8-binding factor (SP8BF), later renamed SWEET POTATO FACTOR 1 (SPF1), was the first WRKY protein identified through a one-hybrid system, using the SP8 element as bait (Ishiguro and Nakamura 1994) (Table 3). EMSA assays showed that SPF1 strongly binds as a monomer to SP8a and weakly to SP8b. Initially, the mechanism by which SPF1 induced *gSPO-A1*, *gSPO-B1* and *gβ-Amy* genes in response to Suc was unclear (Ishiguro and Nakamura 1992, 1994). However, the subsequent identification of SUSIBA2, a SPF1 ortholog in barley (Table 3), provided insights into its transcriptional activation mechanism (Sun et al. 2003). Interestingly, SUSIBA2 does not bind to the SP8a-like element of the *iso1* promoter, but instead it recognizes three A-rich sequences, resembling SURE motifs, along with a nearby W-box (Sun et al. 1999, 2003). Similarly, a sugar-regulatory region from *ClTST2* promoter in watermelon, containing a W-box, was used as bait in a one-hybrid screening, enabling the discovery of SUSIWM1 (Ren et al. 2018) (Table 3). Transient expression assays in watermelon protoplasts confirmed that SUSIWM1 binds to the W-box in the *ClTST2* promoter, as its expression was induced in response to Suc (Ren et al. 2018). Similarly, in pitaya (*Hylocereus polyrhizus*) a W-box was found in the promoters of *HpINV2* and *HpSuSy1*, which encode Suc breakdown enzymes, is recognized by HpWRKY3 (Wei et al. 2019) (Table 3). EMSA and co-expression assays confirmed that HpWRKY3 activates the expression of these genes through the W-box binding (Wei et al. 2019). All these data suggest a conserved role for SUSIWM1 and HpWRKY3 as potential regulators of sugar responses via W-box recognition.

Additionally, the expression of these WRKY TFs is influenced by sugars availability. For instance, *SPF1* is repressed by Suc, whereas *SUSIBA2* is induced (Ishiguro and Nakamura 1994; Sun et al. 2003). On the other hand, *SUSIWM1* and *HpWRKY3* expression showed a positive correlation with soluble sugar levels during fruit maturation. However, further analysis is needed to confirm whether the expression of *SUSIWM1* and *HpWRKY3* is directly induced or dependent on sugar pathways (Ren et al. 2018; Wei et al. 2019).

### bHLH (basic helix-loop-helix) family

One of the largest TF families in plants is the one that features a bHLH domain of approximately 60 amino acids (Riechmann 2002). This domain consists of two distinct regions: a basic domain of 13–17 amino acids that serves as a DNA-binding motif, and a helix-loop-helix (HLH) domain comprising two amphipathic α-helices connected by a loop. The HLH domain mediates protein interactions, allowing the formation of homo- or heterodimers (Riechmann 2002; Feller et al. 2011). Most of bHLH members bind to E-box (*CANNTG*) variants, being the G-box (*CACGTG*) element the most common variant preferred by these TFs. Remarkably, bHLH TFs are involved in a wide range of plant processes, such as hormone signaling, cellular differentiation, flavonoid biosynthesis, abiotic and biotic stress responses, and others (Feller et al. 2011; Seo et al. 2015; Hong 2016).

In relation to sugar responses, the bHLH34 was identified through a one-hybrid screening using the sugar-responsive region of the *AtPGR* promoter as bait. Its close homolog, bHLH104, was subsequently evaluated and also found to regulate *AtPGR* expression in response to Glc in *Arabidopsis* (Min et al. 2017, 2019) (Table 3). Although both bHLHs bind to the G-box element within the regulatory region of *AtPGR*, bHLH34 additionally binds to the *GAGA* core sequence, whereas bHLH104 also interacts with the *AANA* motif. In plants overexpressing *bHLH34* and treated with 6% Glc, the *AtPGR* expression was up-regulated, whereas the overexpression of *bHLH104* resulted in its down-regulation (Min et al. 2017, 2019). Furthermore, overexpression lines of *bHLH34* and *bHLH104* exhibited antagonistic responses to both Glc and ABA; bHLH34 overexpressing plants were hyposensitive, whereas bHLH104-overexpressing plants were hypersensitive (Min et al. 2017, 2019). In contrast, plants lacking these TFs exhibited opposite Glc responses; namely that, compared to the wild-type, *bhlh34* RNAi lines were hypersensitive to Glc, and *bhlh104* mutants were hyposensitive (Min et al. 2017, 2019). In conclusion, regulation of the *AtPGR* by Glc is mediated through bHLH34 and bHLH104 in an antagonistic manner (Chung et al. 2016; Min et al. 2017, 2019). Moreover, since the expression of both *bHLHs* is regulated by Glc and ABA, further studies are necessary to elucidate the intertwined network between bHLHs activation and their downstream effects.

Sugar signaling has also been investigated in apple. In this system, MdHXK1, a homolog of the *Arabidopsis* Glc-sensor HXK1, was found to interact with MdbHLH3 in co-immunoprecipitation experiments, further confirmed by a yeast two-hybrid assays (Hu et al. 2016) (Table 3). Employing transgenic apple calli overexpressing *MdbHLH3*, it was found that MdHXK1 phosphorylates and stabilizes MdbHLH3 upon Glc stimulation, which then induces its binding to activate the expression of anthocyanin-related genes such as *MdMYB1*, *MdANS*, and *MdUFGT* (Hu et al. 2016). As a result, the apple calli exhibited elevated anthocyanin levels. In fact, a similar effect was found in apple fruits transformed with both *MdHXK1* and *MdbHLH3*. On the other hand, gene expression analysis of RNA-seq data obtained from stable transgenic apple fruits overexpressing *MdbHLH3* showed a misregulation of genes associated to carbohydrate metabolism, glycolysis, carbohydrate transport, and fructose 6-phosphate (F6P) metabolism (Yu et al. 2022). Among these genes, the *PYROPHOSPHATE-DEPENDENT PHOSPHOFRUCTOKINASE* (*MdPFPβ*) which was up-regulated, contained on its promoter region multiple G-box-related elements (*CACGTA* and *CACGTC*) (Yu et al. 2022). Binding assays revealed that MdbHLH3 directly binds to these elements within the *MdPFPβ* promoter to induce its expression. The increased expression of *MdPFPβ* resulted in a rise of soluble sugars and photosynthetic rates in fruits and leaves. Such effect was also replicated in tomato overexpressing *MdPFPβ* (Yu et al. 2022). Altogether, these findings indicate that MdbHLH3 is a pivotal element that regulates key fruit components, such as anthocyanin and sugar content, and underscore the functional participation of bHLHs TFs in sugar signaling (Hu et al. 2016; Yu et al. 2022).

### AP2/ERF (apetala 2/ethylene-responsive element-binding protein) family

The AP2/ERF family represents an extensive group of plant-specific TFs that regulate various aspects of plant development, including hormonal and stress responses (Feng et al. 2020). Their DNA binding domain, composed of 60–70 amino acids, adopts a structure with three β-sheet strands followed by an α-helix motif (Feng et al. 2020; Blanc-Mathieu et al. 2024). Based on the presence of additional conserved motifs outside the AP2 domain, the family is divided into three subfamilies: AP2, ERF and DREB (dehydration-responsive element-binding protein) (Blanc-Mathieu et al. 2024). CREs recognized and bound by AP2/ERF TFs vary among these subfamilies (Blanc-Mathieu et al. 2024).

An enhancer activation mutant was isolated by the induction of the reporter *ACTIVATOR of SPO*^*min*^*:LUC 1* (*ASML1*) under low-sugar conditions (Masaki et al. 2005a), corresponding to a mutation in the *WRINKLED* (*WRI1*) gene (Table 3), whose expression is regulated by Suc. The *wri1* mutant showed a disrupted conversion of carbohydrate into fatty acid and triacylglycerol precursors, leading to an increase in soluble sugars and an 80% reduction in seed oil content (Focks and Benning 1998; Ruuska et al. 2002; Cernac and Benning 2004; Cernac et al. 2006). Compared to the wild type, this dysregulation correlated with a decrease in the expression of glycolysis and triacylglycerol biosynthesis genes. Transient expression assays showed that WRI1/ASML1 was responsible for activating *Atβ-Amy*:LUC, suggesting that ASML1/WRI1 may play a role in sugar signaling, regulating carbon flow towards storage oil during seed development (Focks and Benning 1998; Ruuska et al. 2002; Cernac and Benning 2004; Masaki et al. 2005b; Cernac et al. 2006).

The isolation and characterization of four allelic *Arabidopsis* mutants (*gin6*, *sun6*, *sis5* and *isi3*) with reduced sugar sensitivity showed that all corresponded to the *ABI4* gene. The *gin6* mutant was identified given its capacity to grow in high sugar concentrations (330 mM), a condition that inhibits wild-type seedlings growth. Molecular analysis of *gin6* revealed that is allelic to the *abscisic insensitive4* (*abi4*) mutant, highlighting the dual role of ABI4 in sugar and ABA signaling pathways (Arenas-Huertero et al. 2000; Moore et al. 2003). *ABI4* is predominantly expressed in seeds and to a lesser extent in vegetative tissues, with its expression being induced by Glc and ABA (Arroyo et al. 2003; Bossi et al. 2009). In sugar responses, ABI4 acts as an activator for *ABI5*, *SBE2.2,* and its own expression (Arroyo et al. 2003; Bossi et al. 2009) (Table 3), whereas also acts as a repressor of photosynthetic genes like *RBCS* (Acevedo-Hernández et al. 2005). The versatility of ABI4 may depend on interaction with co-regulators, the context of its CRE, or specific post-translational modifications (Gregorio et al. 2014). The maize homolog of ABI4, ZmABI4, regulates the *ADH1* gene by binding to CE1-like in sugar response, and to CE1 element during the ABA response (Niu et al. 2002). Complementation of the *abi4* in *Arabidopsis* with ZmABI4 restored the ABA and sugar responses, demonstrating functional complementarity across species (Niu et al. 2002).

Another maize TF implicated in sugar response is ZmEREB156, whose expression is regulated by sugars (Huang et al. 2016). ZmEREB156 activates the expression of the starch synthesis gene *ZmSSIIIa* by directly binding to its promoter region; however, the CRE involved has not been identified (Huang et al. 2016) (Table 3).

### B3 family

This plant-specific family of TFs possesses a characteristic B3 domain (110 amino acids), that functions as a DNA-binding domain (Swaminathan et al. 2008). Such B3 domain forms a structure with seven β-strands and two short α-helices. The B3 family is subdivided into four subfamilies: ARF (Auxin Response Factor), RAV (Related to ABI3 and VP1), REM (Reproductive Meristem) and LAV (LEC2, Leafy Coyledon2; ABI3; and VAL, VIVIPAROUS/ABI3-LIKE) (Swaminathan et al. 2008; Yamasaki et al. 2013). Within the LAV subfamily are found LEC2, ABI3, and VAL, which recognize the RY element. Besides to the B3 domain, VAL TFs harbor other functional domains, such as an EAR motif, that serves for transcriptional repression, as well as two domains associated with chromatin remodeling, the CW-like zinc finger and PHD-like domains (Swaminathan et al. 2008; Yamasaki et al. 2013).

Members of the VAL subgroup such as VAL1/HSI2 (HIGH-LEVEL EXPRESSION OF SUGAR-INDUCIBLE GENE 2), VAL2/HSL1 (HSI2-like1), and VAL3/HSL2, regulate genes associated with seed development and maturation (Suzuki et al. 1997; Yamasaki et al. 2013). VAL1/HSI2 was initially identified through screening of T-DNA mutants containing the GUS and LUC reporter genes driven by the minimal sugar response promoter of sporamin (Tsukagoshi et al. 2005) (Table 3). The *hsi2* mutant showed increased activities of both reporter genes in the presence of Suc, but it did not exhibit any visible phenotype (Tsukagoshi et al. 2005, 2007). In contrast, the double mutant *hsi2 hsl1* grown in Suc and Glc showed severe alterations, including delayed root hair development, seed oil accumulation, and swollen hypocotyls turning light yellow with a rugged surface, eventually forming a yellow callus-like structure (Tsukagoshi et al. 2007). Microarray analysis of *hsi2 hsl1* growth on Suc revealed misregulation of genes involved in seed development (Suzuki et al. 2007). Based on this, it was concluded that HSI2 and HSL1 function to suppress the expression of embryonic traits after germination. The expression of *VAL1*/*HSI2* and *VAL2*/*HSL1* is induced by Suc, and further studies showed that these TFs have additional roles in other developmental stages. For instance, during the flowering transition phase, VAL1/HSI2 and VAL2/HSL1 interact with components of the POLYCOMB REPRESSIVE COMPLEXES 1, thereby acting as chromatin remodeling proteins (Chen et al. 2022). The motif recognized and bound by these VALs TFs is the RY element. This motif was identified within the cold-responsive regulatory region of the *FLOWERING LOCUS C* (*FLC*) promoter. A one-hybrid assay using this regulatory sequence revealed that VAL1 and VAL2 bind to the RY element, and this interaction was further confirmed by EMSA (Qüesta et al. 2016; Yuan et al. 2016). Consequently, because VAL factors are involved in broader regulatory networks, it has been challenging to assess their specific involvement in sugar signaling responses. Interestingly, the SUC/ROS-3, -4, -5, -8, and -10 elements contain the RY motif (Geisler et al. 2006) (Table 1), suggesting that VAL TFs are indeed involved in the regulation of gene expression by sugars.

### EIN3/EIL (ethylene-insensitive 3/-like) family

This small family, with only six members, exhibits various structural features, including a rich region in acidic amino acids at the N-terminal, followed by a proline-rich region, a coil structure, several highly basic regions, and in some cases, polyasparagine or polyglutamine repeats in the C-terminal region (Li et al. 2019). The acidic, proline-rich, and glutamine-rich regions have been described as the transcriptional activation domains, whereas the coil motif is proposed to be involved in DNA binding and the formation of dimers (Li et al. 2019; Su et al. 2023). Members of this family function as transcriptional regulators in ethylene response (Chao et al. 1997; Li et al. 2019; Su et al. 2023).

Although EIN3 is a key transcriptional regulator in ethylene signaling, its mutation causes hypersensitivity to Glc, whereas its overexpression confers a Glc-insensitive phenotype (Yanagisawa et al. 2003). These findings suggests that EIN3 mediates specific ethylene and Glc responses during plant growth and development (Table 3). It is proposed that a Glc-dependent protein degradation might be the mechanism by which EIN3 exerts its function (Yanagisawa et al. 2003). The close homologue of EIN3, SLIM1, regulates the expression of sulfur metabolism genes under sulfur deficiency conditions, and Glc enhances this regulatory effect. Thus, SLIM1 coordinates sugar signaling with sulfur availability during germination and root growth (Wawrzyńska et al. 2022) (Table 3). The *slim1* mutant displays impaired germination and reduced root growth under sulfur deficiency and high Glc. Expression analysis of genes regulated by the distinct Glc pathways demonstrated that SLIM1 positively participates via a HXK1-dependent pathway during sulfur deficiency. Noteworthy, the expression of *SLIM1* is induced by Glc, independent of sulfur status (Wawrzyńska et al. 2022). In addition, the *slim1* mutant also exhibited altered Suc response, affecting anthocyanin production during sulfur deficiency. In the case of *PAP1,* its expression was reduced by Suc, whereas in wild-type it was induced. Transactivation expression assays in *Nicotiana benthamiana*, demonstrated that SLIM1 binds to the G-box-like sequence (*AGATGCACAT*) within the *PAP1* promoter (Wawrzyńska et al. 2022). Furthermore, mutants in *ETR1*, *EIN2*, and *CTR1*, corresponding to other components of the ethylene signaling pathway, showed altered phenotypes in presence of sugars (Zhou et al. 1998). All these findings support the idea of an interaction between ethylene and sugar signaling, in which EIN3 and SLIM1 are regulatory convergent nodes.

### DUF (domains of unknown function) proteins

DUFs proteins are characterized by a conserved amino acid sequence but lack functional annotation (Bateman et al. 2010). Although diverse methods have been employed to elucidate the roles of DUFs, the functions of many members remain uncharacterized. Among the diverse methods used to characterize DUFs, sequence and structural comparison analysis, inference from genomic data (like STRING and PROLINKS databases), as well as traditional molecular biology techniques (forward genetic, transcriptome analysis, and others) have been employed. Despite these efforts, only a few DUFs have been functionally characterized, highlighting the importance of unveiling their biological roles (Bateman et al. 2010; Lv et al. 2023).

The first DUF protein associated with sugar response was Storekeeper (STK), which was identified through southwestern screening, using the B-box from the patatin promoter gene (Zourelidou et al. 2002) (Table 3). Historically, an unknown protein from potato tuber nuclear extracts was found to bind to a B-box, thereby such protein was named BBBF (B-Box Binding Factor) (Zourelidou et al. 2002). Later, using an antibody assay, the identity of BBBF as STK was confirmed (Zourelidou et al. 2002). STK has a high affinity and binding specificity for the B-box element, notably, its own expression is induced by Suc (Zourelidou et al. 2002). Using the delimited sugar-response region of the *AtPGR* as bait, STK-like1 (AtSTKL1) protein was found in *Arabidopsis* by a one-hybrid system (Chung et al. 2016) (Table 3). Examination of the binding site of AtSTKL1 and its closest homolog, AtSTKL2, revealed the shared consensus core sequence *GCCT*, within the *AtPGR* promoter, with AtSTKL1 displaying a stronger binding affinity to this sequence than AtSTKL2. Furthermore, evaluation of *AtPGR* expression in presence of 6% Glc in AtSTKLs overexpressing or silenced plants, showed that both TFs are transcriptional repressors (Chung et al. 2016). Additionally, *AtSTKL* overexpressing plants were hypersensitive, whereas silenced plants were hyposensitive to Glc (Chung et al. 2011, 2016). Similar phenotypes were observed in tomato plants with altered expression of the *STK* homolog (*SlSTK*). Overexpressor plants of *SlSTK* showed increased Glc sensitivity, whereas *SlSTK* knockout lines were hyposensitive to Glc (Lu et al. 2023). Altogether, these observations suggest that SlSTK function as a negative regulator in the Glc signaling during seed germination (Lu et al. 2023).

### Other TFs involved in sugar signaling

ASML2 is a member of the CONSTANS (CO), CO-like, and TOC1 (CCT) domain family, involved in the regulation of genes associated with photoperiodic flowering, and vernalization (Masaki et al. 2005b). Accordingly, *ASML2* was found expressed in flowers, siliques, steam, and developing seed (Masaki et al. 2005b). Using an activation tagging for reporter genes under the control of the sporamine promoter, ASML2 was identified and showed to function as transcriptional activator of *β-Amy, ApL3*, and *VEGETATIVE STORAGE PROTEIN 2* (*VSP2*) (Masaki et al. 2005b) (Table 3).

Another TF associated with sugar responses is the Homeodomain-leucine zipper (HD-Zip), ATHB13 (Hanson et al. 2001) (Table 3). Transgenic plants overexpressing *ATHB13* developed narrower cotyledons and leaves compared to the wild-type plants. This phenotype became more pronounced as Suc concentrations increased, and a similar effect was observed with Glc, therefore, implying that ATHB13 is involved in the sugar regulation of cotyledon and leaf development (Hanson et al. 2001). In the presence of Suc, expression levels of *β-Amy* and *VSP* were higher in the *ATHB13* overexpressor plants than in the wild-type (Hanson et al. 2001).

In maize, a co-expression analysis of starch synthesis genes and related genes identified the *ZmPLATZ2* TF as their putative regulator (Li et al. 2021) (Table 3). Interestingly, expression of *ZmPLATZ2* is responsive to sugars, being downregulated by Suc and upregulated by Glc (Li et al. 2021). Moreover, through one-hybrid experiments and transient expression assays, it was demonstrated that ZmPLATZ2 binds to the promoters of starch synthesis genes, inducing their expression. Such function was confirmed in rice plants, where *ZmPLATZ2* overexpression, resulted in the induction of *OsSSI*, *OsSSIIIa*, and *OsWX* genes (Li et al. 2021). The A-element was identified as the putative ZmPLATZ2 DNA binding motif by a DAP-seq analysis. Since the A-element was found in the promoter regions of *STARCH SYNTHASE* (*SSI*) genes in maize and rice, it suggests that the mechanism of regulation across species is conserved (Li et al. 2021).

## Synergistic regulation by sugar-responsive CREs and TFs

**Fig. 3:**
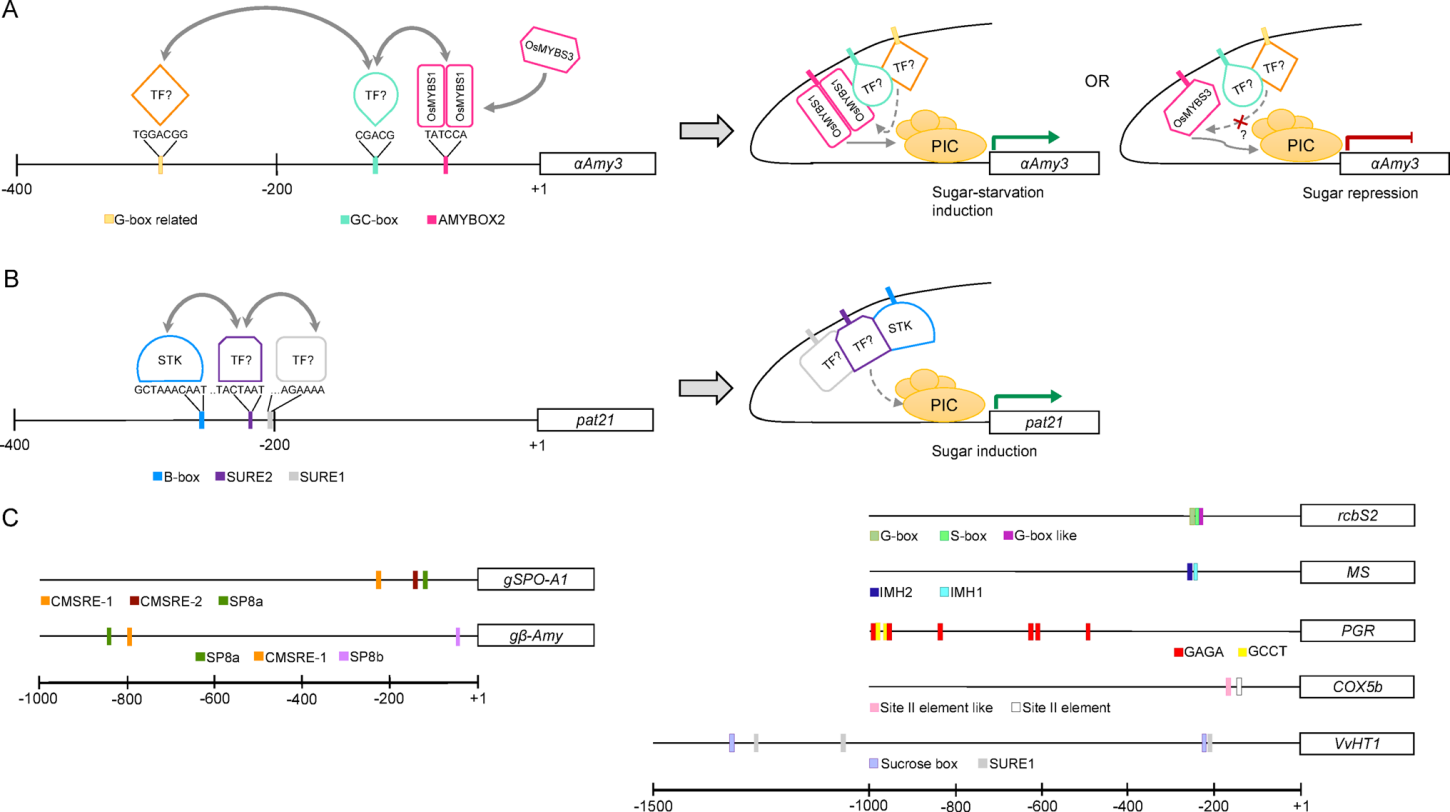
Arrangements of multiple sugar-responsive regulatory elements in some gene promoters. The CREs are indicated by different colors. **A** In *αAmy3,* OsMYBS1 binds to AMYBOX2 as a homodimer to induce expression under starvation, while in the presence of sugar, OsMYBS1 is displaced by OsMYBS3, leading to repression. The TFs binding the GC-box and G-box remain unidentified but are likely part of the sugar response complex (+ 1, relative to transcription start site). **B** The *pat21* promoter contains three CREs, B-box, SURE-1, and SURE-2, that mediate sugar-induced expression. While STK binds to the B-box, the TFs interacting with the SURE elements remain unknown (+ 1, relative to translation start site). **C** Graphic models of diverse arrangements of multiple CREs localized in the promoters of sugar responsive genes, for *gSPO-A1, and gβ-Amy* relative to the transcription site, and *rcbS2, MS, PGR, COX5b* and *VvHT1*, relative to the translation start site

*In vivo*, gene expression is rarely regulated by a single CRE and TF, but rather by the combinatorial action of multiple CREs and the binding TFs that defines whether a gene is ON or OFF. In that sense, studies of gene regulation have emphasized the importance of cooperative interaction, known as synergy, among CREs and TFs in maintaining certain cell states such as cellular identity or responding to developmental and environmental cues. In this way, motif repetition, element order and spacer distances are some factors that can influence a synergistic response (Kawagoe et al. 1994; Carey 1998; Hartmann et al. 2005; Dhatterwal et al. 2019). For example, promoter analysis of sugar-responsive genes has revealed that the expression of *gSPO-A1*,* gβ-Amy*, *pat21*, *αAmy3*, *rbcS2*, *MS*, *PGR* and *VvHT1*, is regulated by multiple sugar CREs (Fig. 3). These sugar CREs may act independently or synergistically to fine-tune expression in response to sugars. For instance, analysis of the *αAmy3* promoter identified the GC-box, G-box and AMYBOX2 as key regulators of starvation-induced expression (Hwang et al. 1998; Lu et al. 1998). When tested separately, the GC-box + G-box combination led to a significant reduction in expression, whereas the AMYBOX2 alone had no effect. However, tandem repeats of AMYBOX2 enhanced transcription beyond that of the GC-box + G-box combination (Lu et al. 1998). Mutational analyses further demonstrated the individual contributions of these elements. Disruptions in the GC-box, G-box and AMYBOX2 reduced expression by approximately 50%, 80% and 90%, respectively, highlighting their synergistic function. EMSA assays confirmed nuclear protein binding to these elements (Lu et al. 1998), and later studies identified that the OsMYBS1 as the TF that binds AMYBOX2 as a homodimer to induce *αAmy3* expression under starvation conditions. In contrast, in the presence of sugars, OsMYBS3 displaces OsMYBS1, and binds to the same element to repress *αAmy3* expression (Fig. 3A) (Lu et al. 2002). However, the TFs targeting the GC-box and G-box remain unidentified but are likely integral to the sugar response complex. Similarly, the promoter region of the *pat21* gene contains three CREs; SURE-1, SURE-2 and the B-box, that mediate its sugar-induced expression. However, their synergistic contribution has not been fully characterized. Whereas the STK has been identified as a B-box binding TF, but the SURE binging TFs have yet to be determined (Fig. 3B) (Grierson et al. 1994; Zourelidou et al. 2002).

Beyond naturally occurring promoters, efforts to engineer synthetic regulatory modules have demonstrated the potential of the cooperative action of CREs to modulate gene expression. Through artificial expression cassettes containing eight common CREs from highly expressed *Arabidopsis* genes, was demonstrated their capacity to active transcription in an individual manner, as well as in a synergistic way, resulting in variable transcription levels of the reporter gene (Sawant et al. 2005). Notably, cooperativity between CREs can form functional regulatory units. That is the case of light regulatory unit, composed of a MYB-recognition site and elements containing *ACGT*-core, which controls some flavonoid biosynthetic pathway genes in *Arabidopsis* (Hartmann et al. 2005). In that sense, sugar CREs may form regulatory units governing sugar response gene expression. Such synergistic combinations of CREs have been identified in several sugar-regulated promoters: GC-box + G-box + AMYBOX2 in *αAmy3* (Fig. 3A); B-box + SURE-1 + SURE-2 in *pat21* (Fig. 3B); CMSRE-1 + CMSRE-2 + SP8a, SP8a + CMSRE-1 + SP8b in *gSPO-A1* and* gβ-Amy*, respectively (Fig. 3C); and G-box + S-box + G-box like, IMH1 + IMH2, *GCCT* + *GAGA* motifs, Site II elements and SURE-1 + Sucrose box 3 in *rcbS2, MS*, *PGR*, *COX5b* and *VvHT1*, respectively (Fig. 3C) (Grierson et al. 1994; Sarah et al. 1996; De Bellis et al. 1997; Atanassova et al. 2003; Comelli and Gonzalez 2009; Mufarrege et al. 2009; Jiaxing et al. 2018). These regulatory modules require further evaluation to clarify their mechanisms and assess their applicability for rational design in gene expression patterns for crop enhancement. Additionally, the possibility of a cooperative effect between CREs located far away cannot be excluded.

The G-box is a highly conserved and widely distributed element in the plant genome. In some genes, G-box mediates responses to both environmental and developmental signals, with its specificity influenced by neighboring CREs. For example, the G-box together with two E-box variant motifs in the *β-phaseolin* promoter, regulates its expression during embryogenesis. In the *rbcS2* promoter of bean, a G-box and a G-box related element (*CACCTG*) form an arrangement that resembles the mammalian ChoRE element (Carbohydrate Response Element), known for its synergistic sugar activation response in primary hepatocytes of rats (Kawagoe et al. 1994; Menkens et al. 1995; Chandrasekharan et al. 2003), however, in *rbcS2*, the repression is the output instead (Urwin and Jenkins 1997). Additionally, an S-box between two G-box elements in the *rbcS2* promoter, suggests that the S-box could be the key element involved in the sugar-mediated repression of *rbcS* genes (Urwin and Jenkins 1997; Acevedo-Hernández et al. 2005).

The spacing between CREs is another key factor for the functional synergistic response. For instance, the evaluation of two copies of the W-box element revealed a gradual reduction in the reporter gene expression as the spacing between the elements increased (Dhatterwal et al. 2019). Similarly, the CMSRE-1 and CMSRE-2 elements require a precise distance of 63 nucleotides for a synergistic sugar response (Morikami et al. 2005). Despite large distances, the flexibility of chromatin allows bending, facilitating the interaction between TFs that bind to distant CREs (Morikami et al. 2005). Interestingly, some sugar CREs, such as SURE-1 and SURE-2, are separated by four nucleotides, as well as the case of Sucrose box 3 and SURE-1, in the promoter of *pat21* and *VvHT1*, respectively (Fig. 3B) (Grierson et al. 1994; Atanassova et al. 2003). On the other hand, tandem arrangements of sugar-responsive CREs are also observed in the promoter of *rbcS2*. Interestingly, a similar pattern of CREs arrangement is found in animal promoters, the ChoRE element requires a specific spacing of five-nucleotide between two E-boxes, whereas a space of 6, 7, or 15 results in a weaker Glc response in some mouse genes (Shih et al. 1995; Poungvarin et al. 2015). Thus, further evaluation of these elements is necessary to determine whether a specific spacer distance is required to modulate gene expression in a synergistic manner in plants.

**Fig. 4:**
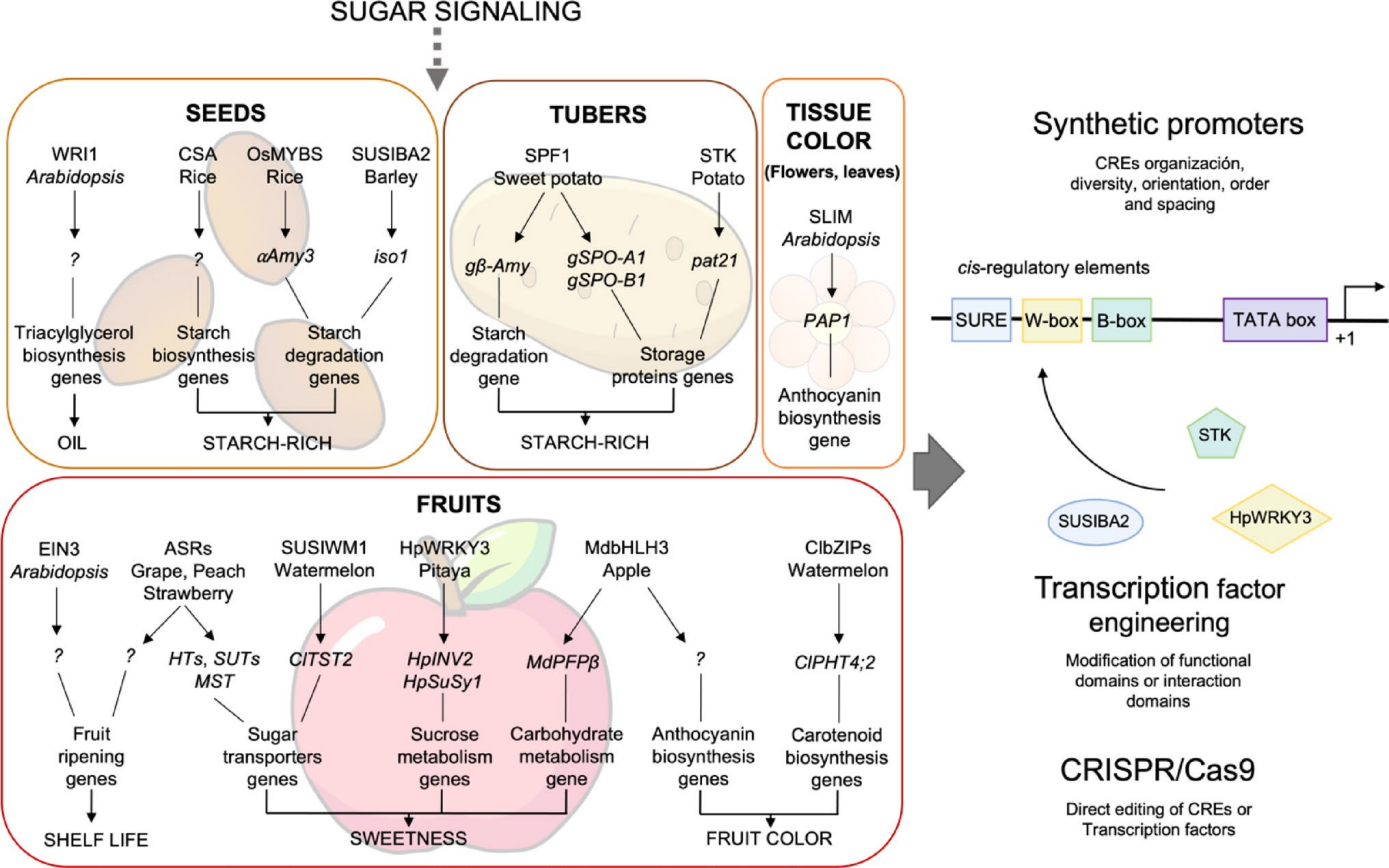
Biotechnological applications of sugar-responsive transcriptional regulation in plants. Sugar signaling networks regulate the expression of certain metabolic and developmental genes in various plant organs, including seeds, tubers, flowers, and fruits. These pathways, mediated by specific transcription factors (e.g., HpWRKY3, SUSIBA2, STK) and sugar-responsive cis-regulatory elements (e.g., SURE, W-box, B-box), control processes such as sucrose metabolism, sugar accumulation, storage compound biosynthesis, and pigment production. Harnessing this regulatory knowledge enables the genetic engineering of commercially valuable traits—such as sweetness, yield, shelf-life and color—in fruits, tubers, and grains. These insights could be used for advanced crop improvement strategies, including the design of synthetic promoters containing sugar-responsive elements, targeted engineering of transcription factors, and the application of genome editing technologies like CRISPR-Cas systems

Genomic blueprints that ensure the proper spatiotemporal patterning of gene expression are encoded by CREs. The combinatorial nature of these regulatory elements creates modules for specific responses or expression in certain tissues, organs, or cells of the plant, such as the radicle, hypocotyl or embryo (Chandrasekharan et al. 2003). Cooperativeness facilitates the assembly of an enhanceosome, a multifactorial complex that involves the sequential binding of various TFs to specific motifs in a predetermined order and orientation (Hartmann et al. 2005; Welchen et al. 2009). Such multifactorial complex regulates the RNA polymerase II activity and/or maintain the complex stability, leading to the proper regulation of gene expression (Carey 1998; Karr et al. 2022). This CREs grammar, namely the order, orientation, spacing, and binding affinity to their corresponding TFs, determines the appropriate gene expression patterns in response to developmental and environmental cues. Understanding this grammar in plants is the first step toward designing synthetic CREs modules to generate genetically improved crops. Deciphering the full network of TF interactions within these regulatory modules is equally crucial.

Whereas techniques such as DAP-seq are invaluable for mapping TF-binding sites in a genome-wide manner, complementary approaches can be used to characterize those protein complexes that mediate gene regulation. Tandem-affinity purification coupled with mass spectrometry (TAP-seq) is one such technique, designed to identify protein interactors within these complexes (Van Leene et al. 2011; Adelmant et al. 2019). TAP-seq involves fusing an affinity tag to a protein of interest, which then serves as bait to isolate associated complexes. The tagged protein is bound to a resin matrix and a cell extract is passed through to capture interacting proteins, which are subsequently identified via mass spectrometric (MS). To enhance purification efficiency, a variety of affinity tags, such as FLAG, HA, MYC, GFP, and biotin have been developed (Kaiser et al. 2008; Van Leene et al. 2011; Adelmant et al. 2019). By tagging known sugar-responsive TFs (such as ABI4, bZIP or MYB factors), TAP-seq could identify novel protein partners that modulate TF activity in response to sugar availability, shedding light on regulatory mechanisms. The reconstruction of these TF complexes will be instrumental in designing synthetic regulatory modules with enhanced precision, ultimately contributing to gene expression regulation in plants.

## Towards biotechnological applications of sugar-responsive transcriptional regulation in plants

Molecular and transformation technologies have been instrumental in developing genetically modified plants with specific characteristics, helping to address agricultural challenges. Initially, emphasis was placed on traits like herbicide and insect resistance in major crops such as maize, soybean, and cotton (Bailey-Serres et al. 2019; Tian et al. 2021). However, consumer preferences have shifted toward traits like flavor, texture, color, and nutritional content, all of which are influenced by physiological and biochemical processes regulated by gene expression (Bailey-Serres et al. 2019; Tian et al. 2021). Extensive research has demonstrated that sugars regulate multiple aspects of plant growth and development including seed, fruit, and tuber formation. In part, this regulation occurs through sugar signaling pathways, where TFs bind to specific CREs within the promoter regions of target genes, activating or repressing their expression (Fig. 4) (Grierson et al. 1994; Cernac and Benning 2004; Durán-Soria et al. 2020). The use of these regulatory elements as tools to control the transcriptional outputs for certain genes represents an excellent approach to exploit their biotechnological potential. One major goal of CRE engineering is to design synthetic promoters that can finely control the expression of transgenes. However, since the native regulation of gene expression relies on precise details of CREs organization, it is crucial to consider factors such as the number (self-repeated or diverse), orientation, order and spacing of CREs. On the other hand, *trans*-factors engineering focuses on modifying TFs by altering functional domains or introducing protein–protein interaction domains to modulate transcriptional activity (Chow et al. 2018; Shrestha et al. 2018; Hummel et al. 2023). Identifying and characterizing these regulatory components in sugar-mediated transcription regulation will facilitate the development of genetically improved vegetable products with enhanced commercial value. The following section discusses selected regulatory elements with biotechnological potential.

Sugars are synthesized in photosynthetic tissues (source) and then transported to non-photosynthetic (sink) organs. In sink organs such as seeds, fruits, and tubers, sugar molecules are accumulated in a soluble form (Glu, Fru and Suc) or transformed in starch or oils (Gibson 2005; Durán-Soria et al. 2020; Aluko et al. 2021). The type of storage molecule depends on the expression of sugar transporter proteins, and biosynthetic or metabolic enzymes, whose gene expression is regulated by sugar-responsive TFs. For example, in *Arabidopsis* seeds, WRI1 regulates the carbon flow toward storage oils by controlling the expression of glycolysis and triacylglycerol biosynthetic genes (Cernac and Benning 2004). Although the specific CRE recognized by WRI1, as well as its target genes, remain unidentified, this TF represents a promising tool for increasing oil accumulation in economically important seeds like those of *Ricinus* (Fig. 4). In rice, particularly three OsMYBS TFs regulate α-amylase gene, whereas CSA controls genes involved in starch biosynthesis (Lu et al. 2002; Zhang et al. 2010). Because the CREs recognized by these TFs have been identified, those CREs are promising candidates for engineering rice, and maize to boost starch-rich yield. Likewise, the TFs SPF1 and STK, which bind to the SP8 and B-box CREs, respectively, regulate the expression of major store protein genes in sweet potato, and potato tubers (Fig. 4) (Ishiguro and Nakamura 1994; Zourelidou et al. 2002). Given the global importance of these tubers, understanding the molecular mechanism involving these TFs is essential.

Transcriptomic analyses have revealed that sugar transporters genes, such as *SUT*s, *SWEET*s, and *MST*s, are responsive to sugar stimuli (Price et al. 2004; Yu et al. 2022). Further studies on *HT1*, *SUT*s, and *TST* indicate that their upregulation is associated with increased sugar accumulation in a variety of fruits (Cakir et al. 2003; Joo and Song 2015; Ren et al. 2018). Notably, altered expression of these transporters impacts fruit ripening (Fig. 4); their overexpression accelerates the process, whereas downregulation delays it. Promoter analyses have identified sugar-responsive elements such as the Sucrose box 3, SURE-1, and W-box in these genes (Atanassova et al. 2003; Jiaxing et al. 2018; Ren et al. 2018). Several TFs that bind to these elements have been identified, with most belonging to the ASR family, which is implicated in sugar-mediated gene regulation (Cakir et al. 2003; Sun et al. 2003; Zhang et al. 2014; Jia et al. 2016; Jiaxing et al. 2018; Parrilla et al. 2022). Sugar availability also regulates the expression of Suc metabolism genes such as *HpINV2* and *HpSuSy1*, which are regulated through the binding of HpWRKY3 to W-box elements in their promoters (Fig. 4) (Wei et al. 2019). Moreover, both Suc and its non-metabolizable analog turanose, have been shown to influence the expression of ripening-related genes and Suc-responsive genes (Jia et al. 2013). Given that growing evidence supports the idea that modulating the expression of sugar transporters can enhance fruit sweetness, these genes are positioned as attractive targets for crop improvement. Collectively, these findings highlight the key role of sugars, particularly Suc, in regulating fruit sweetness and ripening across diverse plant species including grape, strawberry, peach, apple, kiwifruit, melon, and watermelon (Fig. 4) (Cheng et al. 2018; Durán-Soria et al. 2020; Fei et al. 2020).

Fruit color results from the accumulation of anthocyanins and carotenoids, which confer red and yellow-orange tones, respectively, and also serve as important antioxidant agents. These compounds (pigments) are secondary metabolites whose biosynthesis is influenced by several factors including hormones and sugars (Teng et al. 2005; Zhang et al. 2012; Dai et al. 2014; Zhongjie et al. 2015). In *Arabidopsis*, Suc stimulates the flavonoid-anthocyanin pathway, where TFs such as SLIM1 regulates the expression of genes related to anthocyanin biosynthesis and *PAP1* (Hu et al. 2016). In apple, anthocyanin biosynthetic genes are regulated by MdbHLH3 TF (Wawrzyńska et al. 2022). Similarly, Suc promotes carotenoid accumulation by upregulating *PSY*, encoding a rate-limiting enzyme in carotenoid biosynthesis, in juice sacs cultures (Zhang et al. 2012). In watermelon, the expression of the transporter *ClPHT4;2* is regulated by ClbZIP1 and -2, impacting carotenoid content (Zhang et al. 2017). Although much remains to be understood about carotenoid biosynthesis and its regulation by sugars, its relevance extends beyond fruit to ornamental plants, where pigmentation in leaves and flowers can be engineered (Fig. 4). Thus, further molecular insights are need to unlock the full potential of this trait.

Fruits are classified into two ripening groups: climacteric and non-climacteric (Mao et al. 2018; Fei et al. 2020). Climacteric fruits, such as bananas and tomatoes, exhibit a postharvest increased in respiration rate and ripening that is largely regulated by ethylene (Li et al. 2016; Fei et al. 2020). In contrast, the regulatory mechanisms governing the ripening of non-climacteric fruits, including grapes and strawberries, are less well understood, although ABA has been implicated as a key regulator (Mao et al. 2018). Fruit ripening is a complex developmental process coordinated by the interplay of sugar and hormone signaling pathways, largely mediated by their respective CREs in gene promoters. These CREs may act as integrative hubs, translating metabolic and environmental cues into specific gene expression programs. For instance, the regulation of ABA-related genes such as *FaPYR* and *FaNCED* in strawberries, as well as the modulation of ABA levels in grapes, are influenced by Suc, likely through sugar CREs such as Sucrose box 3 and/or SUREs, with ASR TFs serving as key mediators (Cakir et al. 2003; Jia et al. 2013; Mao et al. 2018). Conversely, sugar-associated genes, including sugar transporters, are also responsive to ABA signals. Several TFs involved in sugar signaling can also participate in ABA signaling, and vice versa; this is well exemplified by several ASR and AP2 TFs (Cakir et al. 2003; Bossi et al. 2009; Jia et al. 2016; Jiaxing et al. 2018) (see Table 3). Furthermore, a synergistic effect between sugar and ABA signaling has been observed, particularly in the enhanced accumulation of anthocyanin when both signals are present (Das et al. 2012). Moreover, Suc can activate the expression of ethylene biosynthesis genes like *ACS1* and *ACO2*, thereby promoting postharvest ripening in fruits like tomato and kiwifruits (Li et al. 2016; Fei et al. 2020). EIN3, a key TF in ethylene signaling, was later found to function in sugar responses. Mutants in other ethylene signaling components display altered phenotypes under sugar treatment, further supporting the intertwined relationship roles of sugar and ethylene in plant development and fruit ripening (Zhou et al. 1998; Yanagisawa et al. 2003). Harnessing sugar-responsive elements and their associated TFs offers a powerful biotechnological strategy to precisely control gene expression, enabling improvements in fruit ripening, extended shelf life and postharvest resilience in both climacteric and non-climacteric fruits.

The CRISPR-Cas system is an advanced genome-editing technology that enables precise genetic modifications, thereby accelerating breeding processes without the need for traditional backcrossing (Kafle 2023). It has been employed in resistance breeding, particularly by disrupting susceptibility (*S*) genes to block plant-pathogen interactions. This was achieved by modifying *S* gene promoters or disrupting effector-binding sites to prevent pathogen activation (Zaidi et al. 2018). For instance, knocking out the promoter of *OsSWEET* genes in rice confers resistance to *Xanthomonas oryzae* (Zaidi et al. 2018). Similarly, CRISPR-Cas can be applied to precisely modify sugar CREs and/or their TFs, enabling fine-tuned regulation of sugar metabolism, transport, and accumulation (sugars and/or oils). Such modifications hold the potential to enhance yield, sweetness, coloration, and shelf life. A deeper understanding of the molecular mechanisms through which sugars regulate the development of seed, tuber and fruit (including ripening), has significant implications for genetic engineering aimed at cultivar improvement. Targeting sugar-responsive genes at the transcriptional level offers a promising strategy to enhance crop quality and nutrimental value. As genome-editing technologies continue to evolve, precise manipulation of sugar signaling pathways represents a valuable opportunity to develop high-value cultivars that meet both agronomic and consumer demands.

## Concluding remarks

The function of sugars in plants extends beyond their role as storage molecules for energy and carbon skeletons; they also act as signaling molecules that influence gene expression. This regulation begins with sugar perception and signaling, which influence the activity of TFs that bind to specific CREs within gene promoter regions, ultimately modulating gene activation or repression. Unraveling these regulatory mechanisms has been of great importance in plant biology, as it provides insight into the complex molecular networks governing plant growth and development. The molecular techniques and approaches applied to investigate and understand sugar sensing, signaling and response in plants have evolved significantly, shifting from single gene analyses to genome-wide approaches. Whereas this review presents a comprehensive overview of the known *cis-* and *trans*-acting elements involved in sugar signaling, it also acknowledges the dynamic nature of research in this field, with new findings continually emerging and providing an integral view of sugar regulation of gene expression.

Importantly, this knowledge holds great promise for improving key plant traits such as seed and fruit development, sweetness, and postharvest quality. By leveraging sugar CREs and their associated TFs, it is possible to develop biotechnological strategies aimed at fine-tuning sugar metabolism and accumulation. Precision modulation of sugar signaling pathways, especially through genome edition technologies, offers exciting opportunities to improve crop yield, flavor, color, and shelf life. As research continues to advance, integrating this molecular knowledge into breeding programs and biotechnological applications will be essential for optimizing crop performance and addressing the evolving demands of agriculture. Ultimately, understanding sugar signaling at the molecular level is not only a fundamental pursuit in plant science, but also a strategic avenue toward sustainable and innovative agricultural solutions.
